# Parental Contributions to Offspring Skin Microbiota Remain Detectable After Metamorphosis

**DOI:** 10.1111/mec.70660

**Published:** 2026-11-13

**Authors:** Francesca N. Angiolani‐Larrea, Rayan Bouchali, Adeline Loyau, Dirk S. Schmeller, Eva Ringler

**Affiliations:** ^1^ Division of Behavioural Ecology, Institute of Ecology and Evolution University of Bern Bern Switzerland; ^2^ Fundación Cóndor Andino Ecuador Quito Ecuador; ^3^ Centre de Recherche sur la Biodiversité et l'Environnement (CRBE, UMR 5300) Université de Toulouse, Toulouse INP, CNRS, IRD, CRBE Toulouse France

**Keywords:** cross‐foster experiment, microbial transmission, skin microbiome, social interactions, tadpole transport

## Abstract

Parental care creates opportunities for microbial transmission, yet its contribution to offspring skin microbiota remains poorly understood, particularly in species undergoing metamorphosis. We investigated this role in the Neotropical poison frog *Allobates femoralis* using a controlled cross‐fostering experiment. Across 30 trials, tadpoles were transported by their biological father, a foster father or transferred manually to a provided water bowl. We characterized bacterial communities in adults, clutch mucus, tadpoles, metamorphs and environmental samples using 16S rRNA gene sequencing, Bayesian source tracking and analyses of shared sequence variants. Source tracking estimated contributions from both biological parents and, in cross‐fostered offspring, foster fathers, alongside environmental sources. Parental microbial contributions remained detectable after metamorphosis despite substantial changes in community composition, indicating that early parental contact may influence microbial assembly across developmental stages. Because foster fathers contacted clutches before transport, their contributions could reflect microbial transfer during brooding, transport or both. Some sequence variants shared between fathers and offspring belonged to genera containing documented *Batrachochytrium dendrobatidis*‐inhibitory isolates, although neither inhibitory activity nor disease protection was tested. Our findings identify paternal care as a pathway contributing to offspring skin microbial assembly under laboratory conditions. They extend the significance of parental care beyond provisioning and relocation, while highlighting the need to establish taxon‐level persistence, functional consequences and transmission dynamics in natural populations.

## 1 Introduction

Social behaviour plays a pivotal role in the transfer of various materials between individual animals (Hakala et al. 2022). For example, during mating, grooming or parental care, social partners or parents and offspring stay in close spatial association with each other, which facilitates the transfer of chemicals, proteins, nutrients, as well as microbiota (Archie and Tung 2015; Walke et al. 2011). Most organisms are associated with high numbers of symbiotic microorganisms which live both inside and outside of their body (Obeng et al. 2021; Ross et al. 2019; Ursell et al. 2012). These microbiomes have been shown to hold key roles in the host health, as they stimulate or are a direct part of the immune system (Muegge et al. 2011; Yatsunenko et al. 2012; Loyau et al. 2024; Ito et al. 2024). However, we have limited knowledge about the role of social interactions contributing to the building and development of the microbiome, especially in species with complex life cycles (Valles‐Colomer et al. 2022; Raulo et al. 2021; Pearce et al. 2024). In humans, infants acquire gut microorganisms through exposure during birth and breastfeeding (Praveen et al. 2015; Ursell et al. 2012; Ferretti et al. 2018). In other animals microbial transfers between parents and offspring have mainly been found in species where parents feed their young, such as mucus‐feeding fish (Sylvain and Derome 2017), pigeons that feed on crop milk (Ding et al. 2020) or skin‐feeding caecilians (Kouete et al. 2023), as well as social insects (Russell and Moran 2005; Capodeanu‐Nägler et al. 2018; Körner et al. 2023).

The skin is essential to host health, as it acts as a physical protective barrier against external threats such as pollutants or pathogens, and is associated with diverse microorganisms (Ross et al. 2019). In amphibians, the skin is of particular importance as it regulates key body functions such as respiration, hydration and immune responses (Woodhams et al. 2023; Hillyard 1999; Larsen 2021). Diversity of the amphibian skin microbiome leads to increased protection from lethal fungal pathogens, such as *Batrachochytrium dendrobatidis*, *Batrachochytrium salamandrivorans* (Muletz‐Wolz et al. 2017; Jiménez et al. 2022; Piovia‐Scott et al. 2017; Harris et al. 2009) or ranaviruses (Harrison et al. 2019), through metabolites produced by bacteria (Woodhams et al. 2023) or direct competition between the pathogen and bacteria (Bernardo‐Cravo et al. 2020). Empirical evidence for direct transfer of skin microbiota through contact between amphibian adults and offspring remains limited (McGrath‐Blaser et al. 2021; Fischer et al. 2025; Kouete et al. 2023; see also Hughey et al. 2017). Many amphibian taxa undergo dramatic morphological and physiological changes during metamorphosis (Brown and Cai 2007), which are known to alter skin microbiome composition (Martínez‐Ugalde et al. 2022; Hartmann et al. 2023). These shifts may limit the long‐term effectiveness of early microbial seeding. While pronounced microbiome restructuring has been observed across developmental stages (Kueneman et al. 2014; Prest et al. 2018; see also Kohl et al. 2013), it remains unclear whether microbiota acquired via social contact in earlier stages persists through and beyond metamorphosis.

Amphibians exhibit diverse reproductive modes (Schulte et al. 2020; Ringler et al. 2023), which offer a unique opportunity to study the role of social behaviours for microbiota acquisition. In Neotropical poison frogs, parental care is obligate for offspring survival, as clutches are deposited in the leaf litter or on other terrestrial structures, and the hatched tadpoles are then transported on the back of a parent to water bodies where their development is completed (Ringler et al. 2013; Schulte and Mayer 2017; Weygoldt 1987; Summers and Tumulty 2014). Tadpole transport has long been viewed as a behavioural adaptation to assure that terrestrial eggs develop into aquatic larvae (Ringler et al. 2023). During tadpole transport, prolonged skin‐to‐skin contact between fathers and offspring may facilitate skin microbiome transmission. By combining experiments in the field as well as in the lab, a previous study found that substantial bacterial colonization of embryos takes place directly after hatching, and that tadpole transport indeed serves as a source of skin microbes for transported tadpoles (Fischer et al. 2025). After 4 weeks, transported tadpoles retained isolated bacteria of their caretaker's core community. However, that study did not find a higher degree of similarity between microbial communities of tadpoles and adults in species that transport their offspring compared to those that did not. Communities of tadpoles also did not show a higher similarity to their caregiver than to unrelated adults, suggesting that most caregiver‐associated microbes do not remain in tadpole communities long‐term. While this study provides first compelling evidence of parental transmission of skin microbiota in a poison frog, the question remains whether transferred microbiota are maintained beyond metamorphosis.

We used a cross‐fostering experiment in the Neotropical poison frog *Allobates femoralis* to test how paternal care contributes to offspring skin microbiota. Under controlled laboratory conditions, hatched tadpoles were transported either by their own father, by foster fathers or manually transferred to the provided water bodies, to identify the role of tadpole transport in microbial transfer from adult frogs to offspring. All tadpoles were reared beyond metamorphosis to identify if acquired microbiota are indeed retained until this developmental stage, and to reveal potential benefits to microbial composition (e.g., persistence of protective microbes).

Our experimental setup allowed us to assess microbial contributions from biological and foster parents to offspring over time. We found a clear microbial contribution from the biological parents, as well as the foster father in the cross‐foster treatment. These microbial communities were detected beyond metamorphosis, despite significant shifts in relative abundance. The microbial contribution by parents included several bacterial genera that were previously shown to include strains that are effective against the detrimental amphibian pathogen *B. dendrobatidis* (*Bd*), highlighting the diverse functional benefits of close parent‐offspring interactions.

## 2 Materials and Methods

### 2.1 Study Species and Lab Population

The Neotropical poison frog *A. femoralis* has a pan‐Amazonian distribution with isolated local populations. During the reproductive season, males call from elevated structures on the forest floor to announce their territory to male competitors and to attract females (Roithmair 1992; Ringler et al. 2009). Pair formation, courtship and mating take place in the male's territory (Ringler et al. 2012; Stückler et al. 2019; Fischer et al. 2020; Montanarin et al. 2011), where externally fertilized clutches are deposited in the leaf litter. Males occasionally visit clutches and hydrate them in cases of low humidity (E.R. pers. observation). Tadpole transport to water bodies takes place after 15–20 days of larval development, is mainly performed by males after heavy rains (Aichinger 1991), and usually lasts a few hours (Ringler et al. 2013). In the wild, *A. femoralis* uses diverse water bodies ranging from floodplains to medium sized temporal pools for tadpole deposition (Ringler et al. 2009, 2015; Fouilloux et al. 2021).

We conducted our study in 2022 under controlled laboratory conditions in the animal care facilities at the Ethological station from the University of Bern, Switzerland. Housing conditions are described in the Supplementary Material File 1 (SM1).

### 2.2 Cross‐Foster Experiment and Microbiome Sampling

We initiated a total of 52 trials, of which 22 could not be considered in the final analysis because of developmental issues (e.g., the clutch did not hatch or the tadpoles died early in the experiment), leaving 30 successful trials. Those trials included 23 adult males and 25 adult females in different combinations as breeding pairs. A cleaned and autoclaved water bowl was placed inside the tanks and filled with demineralized water at the start of each trial. Once a breeding pair laid a clutch, we removed the female from the tank and randomly assigned the clutch to one out of three treatments (Figure 1): In the ‘normal transport’ treatment (*N* = 9), the male had full access to its own clutch and was allowed to transport the tadpoles to the provided water bodies inside its tank. Similar to natural conditions, tadpole transport in the lab takes a few hours and is usually performed during morning hours (unpublished data). This treatment was used as a positive control. In the ‘no transport’ treatment (*N* = 9), a net cage was used to prevent further physical contact between the male and the clutch. After hatching, the tadpoles were manually transferred to the provided water bowl by placing the leaf where the clutch was deposited. This treatment was used as a negative control. In the ‘foster transport’ treatment (*N* = 12), we exchanged a recently laid clutch (1–3 days after oviposition) with a stage‐matched clutch of another breeding pair. To do so, clutches were moved together with the leaf they were laid on. New gloves were always used when transferring the clutches or tadpoles to water or between tanks to avoid cross contamination with human skin microbiome. Like in the ‘normal transport’ treatment, the foster father had full access to the cross‐fostered clutch.

**FIGURE 1:**
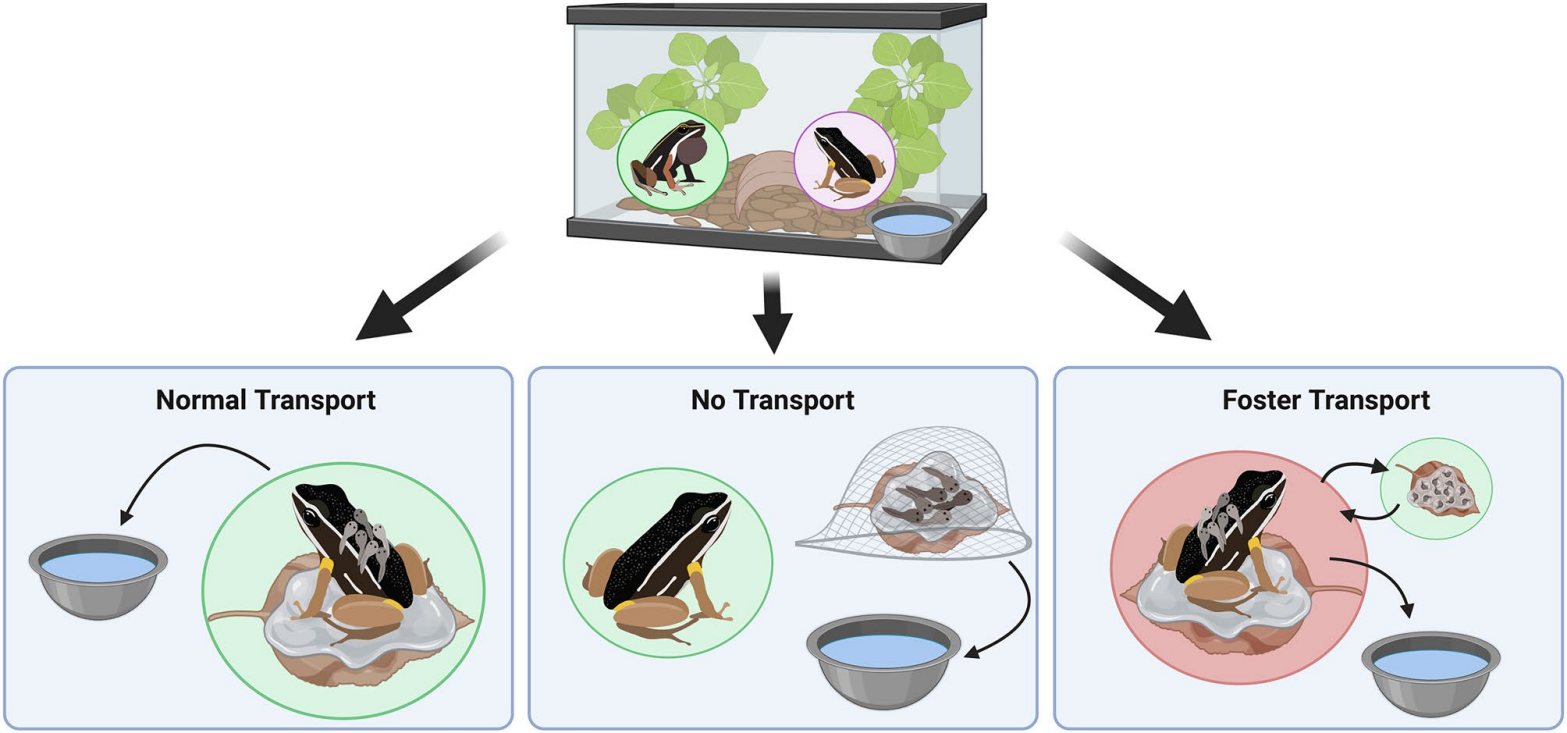
Experimental design. Initial setup composition of all trials with adult male and female frogs cohabiting in a tank with substrate (leaves and pebbles), a water bowl and a shelter. The experimental design comprises three treatments, where in the ‘normal transport’ treatment, tadpole transport is performed by the biological fathers; in the ‘no transport’ treatment, tadpole transport by the father was prevented by placing a net cage over the clutch; and after hatching, tadpoles were manually transferred to the water bowl by experimenters; and in the ‘Foster transport’ treatment, clutches were exchanged between tanks and thus foster fathers transported the focal tadpoles.

All clutches were closely monitored until hatching. After tadpoles had been deposited in the water bowls (by frogs, or by manual transfer in the ‘no transport’ condition), we poured the water and tadpoles into separate bigger aquaria (20 × 20 × 40 cm) to rear all tadpoles clutch‐wise and separate from the tank environment. After metamorphosis (approx. 2 months after transfer), juvenile frogs were moved by clutch groups to rearing enclosures equipped with autoclaved pebbles until final microbiome swabbing (for approx. 2 weeks after metamorphosis).

For microbial sampling, all adults were caught at the onset of each trial with transparent plastic bags, and a new bag and new gloves were used for each frog to avoid cross‐contamination. We collected two swabs per individual, one from the ventral abdominal skin and one from the dorsal skin. To obtain information on the environmental microbiome, we swabbed the substrate (leaves and pebbles) inside each tank and dipped swabs in water bowls and lightly scraped the bottom (i.e., tank samples). Adult skin microbiome was sampled a single time to minimize stress of handling for the individuals, even if the same individual was used in different trials (e.g., as biological father in one and foster father in another trial). We consider the single skin swabs for adult individuals to be representative, because of the short difference in time between trials and the controlled laboratory conditions, as opposed to natural conditions where time differences have proven to change composition significantly (Ellison et al. 2021). Microbial samples of clutch‐associated mucus were collected after the first (w1) and second (w2) week after oviposition by carefully swabbing the surface of the clutch and slightly dipping the swab tip into the egg jelly envelope (subsequently referred to as ‘clutch samples’). Microbial samples of tadpole skin were collected by gently swabbing over the main body and tail of tadpoles (up to 5 tadpoles per clutch). Sample collection took place once the tadpoles were big enough to withstand swabbing (at least 1 cm long from mouth to tail tip, corresponding to about 1 month old tadpoles). Finally, we swabbed the dorsum of available metamorphs per clutch, once they reached at least 1 cm of snout‐vent‐length (about 1 week after metamorphosis). All samples were taken using sterile swabs (COPAN, Medical Wire MW100 Swab) and immediately stored at −20°C until DNA extraction.

### 2.3 DNA Extraction, Sequencing and Bioinformatics

DNA was extracted from swabs using the Macherey‐Nagel NucleoSpin Soil kit (Valencia, CA, USA) according to the manufacturer's protocol, with lysis Solution 2 (SL2).

We prepared the libraries targeting the 16S rRNA gene at the Genetic Diversity Centre (GDC) from ETH Zurich through a two‐step PCR protocol (see SM1 for more details). For the first step PCR, we used primers with overhanging adaptors (381F: TCGTCGGCAGCGTC‐AGATGTGTATAAGAGACAG‐[N]3‐AG‐CCTACGGGNGGCWGCAG; 785R: GTCTCGTGGGCTCGG‐AGATGTGTATAAGAGACAG‐[N]3‐CC‐GACTACHVGGGTATCTAATCC designed at the GDC and adapted from Klindworth et al. (2013)). Demultiplexing, filtering and clustering service was provided by the GDC with the UNOISE pipeline (Genetic Diversity Center 2026) and the SILVA SSU v138 reference (Quast et al. 2013) was used for taxonomic affiliations.

Data analyses were conducted in R 4.4.0 (R Core Team 2024), with RStudio 2024.04.2 (RStudio Team 2024). We used zero‐radius Operational Taxonomic Units (zOTUs) rather than traditional 97% OTUs for all of our analyses, to maximize taxonomic resolution and ensure that patterns of microbial transmission and persistence reflected genuine biological variation rather than clustering artefacts, while also enabling direct comparability with other studies using exact sequence variant approaches. zOTUs are exact sequence variants inferred after denoising, with each zOTU representing a unique sequence (100% sequence identity). We rarefied all samples for all downstream analyses (see below). To determine differential compositions between dorsal and ventral samples from the same frogs, we used the ‘vegan’ package (version 2.6–6.1; Oksanen et al. 2026). We performed a Nonmetric Multidimensional Scaling with Stable Solution from Random Starts (metaMDS function) paired with an ANOSIM test (*anosim* function). We used rarefied samples and Bray–Curtis distances using 999 permutations (*rarefy* and *vegdist* functions, more details are provided in the SM1). We did not find compositional differences between ventral and dorsal samples of the same adult frogs (ANOSIM = −0.01; *p* = 0.389), therefore, in rare cases (*N* = 5) we replaced dorsal samples that were not retained after filtering (see below) with their ventral counterparts.

After sequence filtering, the dataset comprised samples from 23 adult males, 25 adult females, 22 clutches at Week 1, 25 clutches at Week 2, 85 tadpoles and 55 metamorphs, together with nine substrate samples and 21 water samples. These samples represented at total of 30 trials. We use ‘males’ for all adult males in the study and ‘biological fathers’ or ‘foster fathers’ for their roles in individual trials.

### 2.4 Statistical Analyses

To standardize sequencing depth, we rarefied eligible samples to 700 reads per sample. We compared results from this dataset with those obtained using more restrictive filtering criteria; the main findings were similar (see SM1, Table S1). We used the dataset retaining more samples for the analyses reported here. We applied a Hellinger transformation to zOTU abundances and visualized differences among sample types using non‐metric multidimensional scaling (NMDS; Figure 2A). We tested overall differences using ANOSIM on Euclidean distances and then performed pairwise comparisons (Table S2).

**FIGURE 2:**
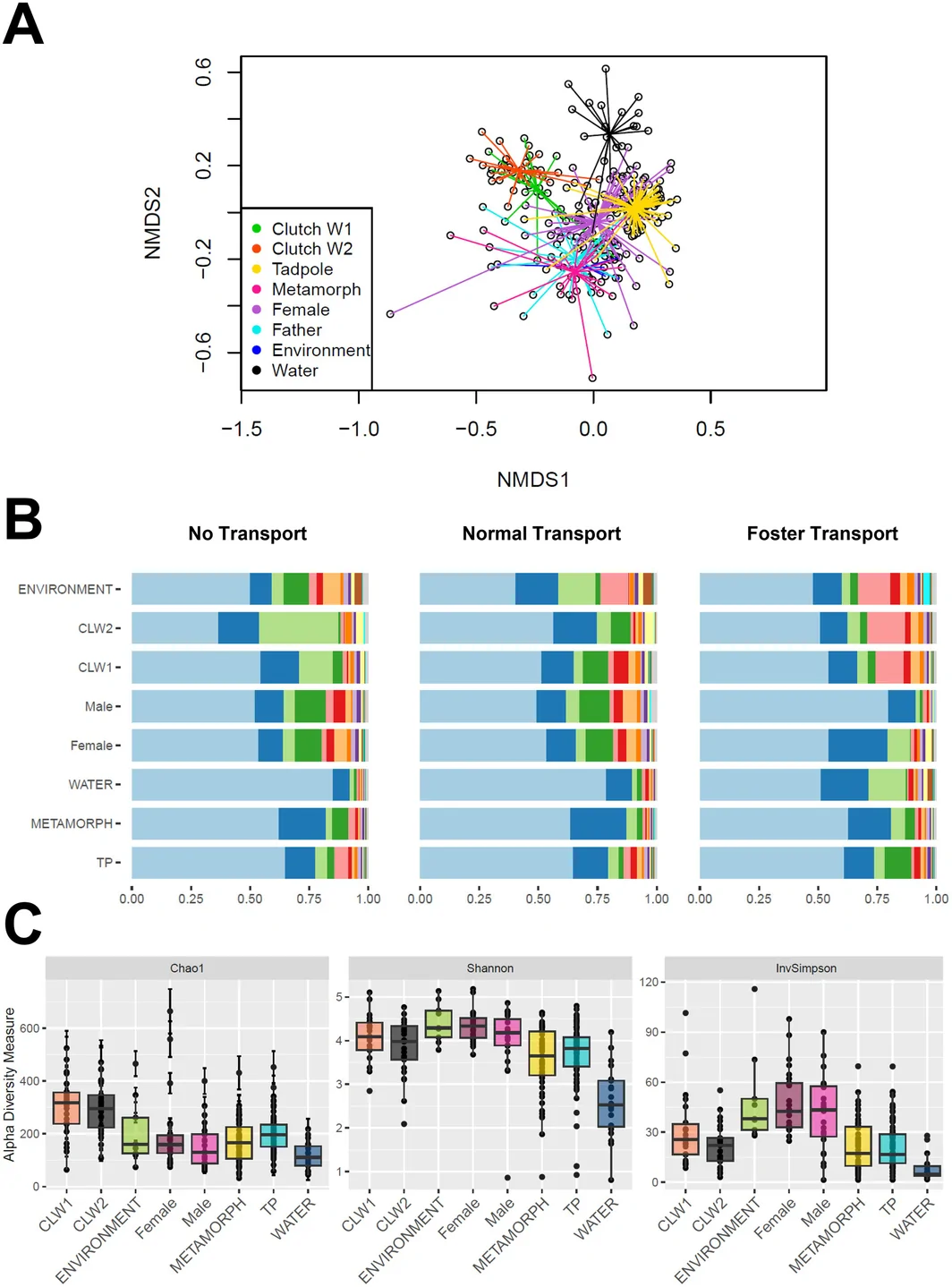
(A) Non‐metric multidimensional scaling (NMDS) ordination plot of the microbial community found in our experiment clustered by sample types (Euclidean distance, *k* = 2, stress = 0.1), (B) abundance of microbiome composition by sample types grouped by Phylum, (C) calculated alpha diversity indices (Chao1, Shannon and Inverse Simpson) of the different sample types.

To test for differences in microbial alpha‐diversity between sample types across treatments, we computed Shannon (richness and evenness, sensitive to rare taxa), Chao1 (total taxon richness) and Inverse Simpson (richness and evenness weighted for dominant taxa) indices and tested for differences with either ANOVA or Kruskal–Wallis tests followed by post hoc tests when necessary (Tables S3 and S4). To evaluate the number of zOTUs that were shared between sample types, we created intersection plots separately by treatment and by trial (see Figures S1 and S2 in SM1). Moreover, we extracted the shared zOTUs from each of the trials by computing a presence‐absence formula (see SM1).

To trace microbial acquisition from the various sources to offspring, we used the Bayesian source‐sink tracking tool SourceTracker2 as it considers the entire microbial community profile in complex mixtures. SourceTracker2 does not require source communities to be statistically different in their overall taxonomic composition. Rather than relying on a prior test of community dissimilarity, the Bayesian framework estimates the probability that each ASV in a sink sample originates from each potential source based on the taxonomic distributions learned from the source communities (Knights et al. 2011). Our different sources were significantly distinct (ANOSIM *R* = 0.54; *p* = 0.001), further supporting the use of SourceTracker2. To test for differences in the percentage of contribution of each source between experimental conditions, we used a generalized linear mixed effects model with the medians of the proportion of contribution from sources as the response variable, treatment, sink and source as fixed effects, and trial identity as a random effect. To determine the differences in contribution between biological and foster fathers, we used a generalized linear mixed model (GLMM) built only with the foster transport treatment trials, with the medians of the proportion of contribution as the response variable, source and sink as fixed effects and trial identity as a random effect. To determine the exact acquisition through tadpole transport, we calculated the zOTUs shared between fathers and tadpoles per experimental condition (normal transport, no transport, foster transport). We also used SourceTracker2 to closely follow the changes in skin microbial composition throughout development. We tested the differences in the proportion of contribution with a generalized linear mixed effects model. We used the proportion of contribution from sources as the response variable, treatment and sink as fixed effects and trial identity as a random effect.

To determine if there were differences in the acquired taxa linked to the treatments, we used a generalized linear model where the number of zOTUs shared between fathers and offspring is set as the response variable and independent variables are the treatment and offspring type (i.e., tadpole or metamorph); trial was included as random factor. To identify which putative *Bd* inhibitory taxa were acquired by the offspring from adults during tadpole transport, we compared our full microbiome dataset with the reference list reported earlier (Loyau et al. 2024). We then compared the zOTUs within tadpole and metamorph samples for each treatment to this list. To determine if the acquisition of putative *Bd* inhibitory taxa was a non‐random process, we used a generalized linear mixed effects model to compare the proportion of putative *Bd*‐inhibitory taxa acquired by offspring of the foster transport treatment from the foster versus biological fathers. We used number of putative inhibitory zOTUs as response variable, source and offspring as independent factors and trial as random factor. We diagnosed our models and tested for significance of independent variables with an Analysis of Deviance (Type II Wald chi square test). More details on the methods are provided in SM1.

## 3 Results

We found significant differences in microbial composition across sample types (ANOSIM *R* = 0.68; *p* = 0.001; Figure 2A,B). The pairwise comparisons confirmed that most sample types differed significantly from each other (*p* < 0.05, see SM1 for the detailed results). We did not find significant differences in microbial composition between male and female adult samples (ANOSIM *R* = −0.02; *p*‐adj = 1), nor between adult samples and the substrate (males vs. substrate: ANOSIM *R* = −0.11; *p*‐adj = 1; female vs. substrate: ANOSIM *R* = −0.12; *p*‐adj = 1). Moreover, the composition of clutch‐associated microbiota from Week 1 and Week 2 (ANOSIM *R* = 0.16; *p*‐adj = 0.056) and of substrate and metamorphs (ANOSIM *R* = 0.37; *p*‐adj = 0.056) did not differ significantly from each other. A detailed description of the microbial composition of different sample types is given in the SM1.

Overall alpha‐diversity differed significantly among sample types (Kruskal–Wallis: Chao1, *χ* ^2^ = 71.41, df = 7, *p* < 0.01; Shannon, *χ* ^2^ = 72.37, df = 7, *p* < 0.01; Inverse Simpson, *χ* ^2^ = 87.16, df = 7, *p* < 0.01; Figure 2C). We therefore conducted pairwise comparisons between sample types. Substrate and adult samples did not differ significantly from one another, nor did adult male and female samples. Similarly, tadpole and metamorph microbial diversity did not differ significantly. Additionally, treatment had no significant effect on offspring alpha‐diversity (see SM1 for details, Figure S3).

### 3.1 Sources of Microbial Acquisition

We found a higher number of shared zOTUs between the tadpole and metamorph samples (normal transport: 89 shared zOTUs; no‐transport: 112 shared zOTUs; foster transport: 105 shared zOTUs, see also SM1) than the rest of all possible combinations. Maternal samples shared 26 zOTUs with tadpole samples in the normal transport treatment, 25 in the no‐transport treatment and 29 in the foster treatment. Adult male samples had 38 shared zOTUs with tadpoles but no detectable shared zOTUs with the metamorphs in the no‐transport treatment. In turn, there were clearly detectable shared zOTUs between adult males and tadpoles or metamorphs in the normal transport (tadpoles: 25 shared zOTUs; metamorphs: 21 shared zOTUs) and foster transport (tadpoles: 37 shared zOTUs; metamorphs: 33 shared zOTUs) treatments. We found no detectable overlap between water and tadpole or metamorph samples in the normal or foster transport treatments; however, we found 32 zOTUs shared only between water and tadpole samples and 23 zOTUs shared between water, tadpole and metamorph samples in the no transport treatment. zOTUs present in substrate samples were present in all other sample types across treatments (normal transport: 41; no transport: 46; foster transport: 70).

The SourceTracker analysis identified substrate, water and both parents, as well as foster fathers in the foster treatment, were all sources for the acquisition of skin microbiota in offspring (Figure 3; Table S5), despite significant differences in the sources' relative contribution (Anova.glmmTMB *X* ^2^ = 31.75, df = 4, *p* ≤ 0.001; Table S6; see SM1 for pairwise comparisons).

**FIGURE 3:**
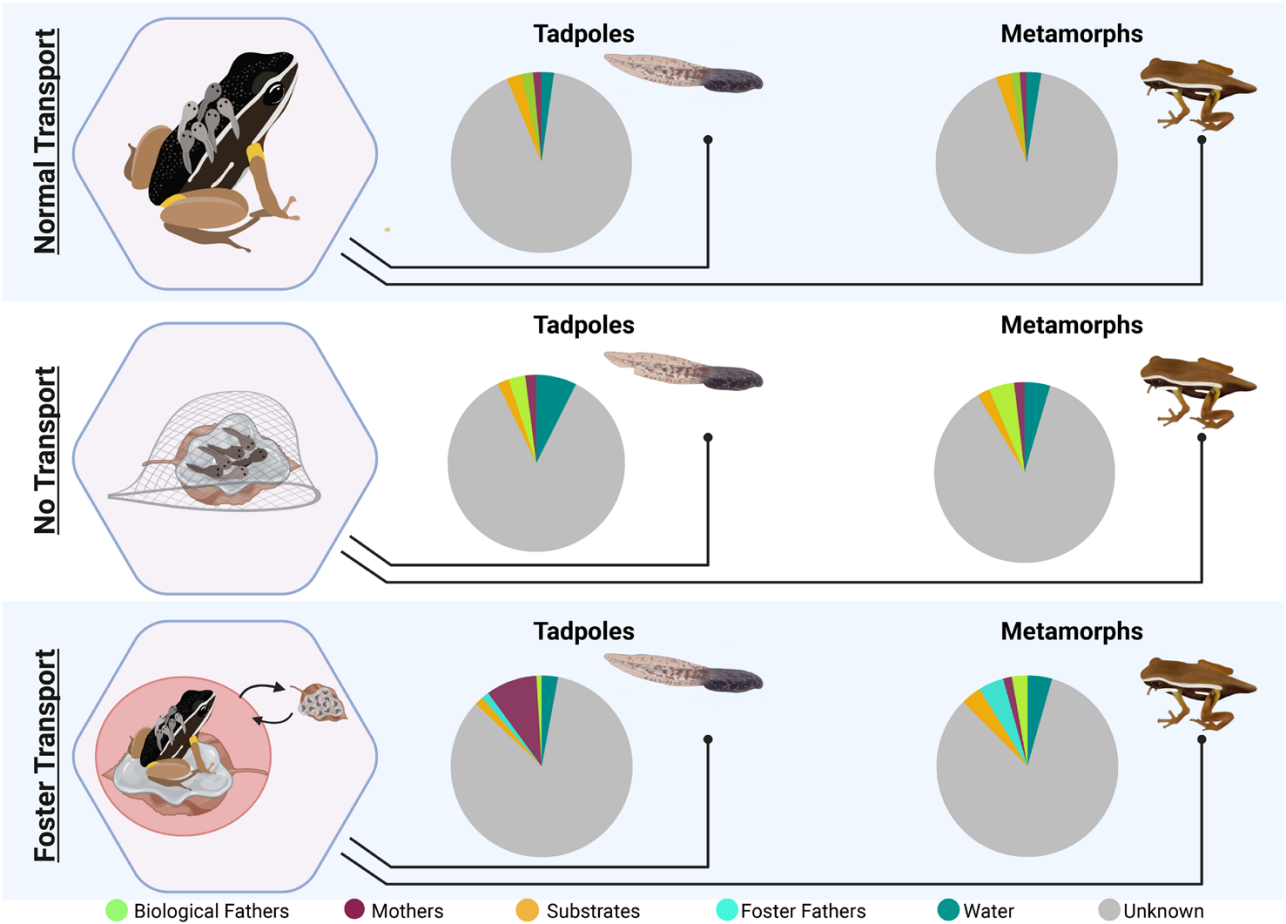
Average relative contributions of all tested sources to tadpole and metamorph samples as determined by the SourceTracker2 analysis. Pie charts show the microbial contribution from each source to the respective offspring stages.

A large part of the microbiome was changing between developmental stages, regardless of treatment (treatment: Anova.glmmTMB *X* ^2^ = 1.78, df = 2, *p* = 0.411; developmental stage: Anova.glmmTMB *X* ^2^ = 138.99, df = 2, *p* < 0.001). We found that hatching was the stage with significantly less microbial contribution from source to sink (clutch‐associated microbiota—tadpole: *β* = 3.45, SE = 0.34, *z* = 10.19, *p* < 0.001; tadpole—metamorph: *β* = −1.82, SE = 0.23, *z* = −7.91, *p* < 0.001; clutch‐associated microbiota—metamorph: *β* = 1.62, SE = 0.37, *z* = 4.41, *p* < 0.001). The change in composition of clutch‐associated microbiota was most variable across treatments (contribution from clutches w1 to clutches w2: normal transport: median = 3.94% (7.40 IQR); no transport: 8.87% (61.83 IQR); foster transport: 9.06% (20.85 IQR)). While the transfer of clutch‐associated microbiota to tadpoles was very low (normal transport: 0.56%  (0.63 IQR); no transport: 0.71%  (0.61 IQR); foster transport: 0.69% (0.77 IQR), Figure 3), the maintenance of the microbiome from tadpoles to metamorphs was comparatively high (normal transport: 2.15% (1.99 IQR); no transport: 2.41%  (1.53 IQR); foster transport: 3.73% (6.21 IQR), see Supplementary Material File 2). We identified significant deviations from model assumptions (overdispersion: *p* = 0.016), however, this can be explained by the large sample size of our dataset and the sensitivity of the DHARMa test (Hartig 2016). Visual inspection of the QQ plot residuals suggests that the distribution of residuals is very close to the expected optimal values (see SM1, Figure S4).

### 3.2 Putative *Bd*‐Inhibitory Taxa Acquired

Using a pairwise presence–absence approach, we identified zOTUs shared exclusively between adult males and their corresponding offspring samples. We found that in the normal transport treatment, biological fathers shared 13 zOTUs with their tadpoles (including 1 putative *Bd*‐inhibitory taxon) and 11 zOTUs with their metamorphs (2 putative inhibitors). In the no‐transport treatment, biological fathers shared 29 zOTUs with tadpoles (8 putative inhibitors) and 12 zOTUs with metamorphs (3 putative inhibitors). In the foster transport treatment, foster fathers shared 20 zOTUs with tadpoles (3 putative inhibitors) and 30 zOTUs with metamorphs (3 putative inhibitors). The putative *Bd*‐inhibitory taxa included the genera *Acinetobacter*, *Alishewanella*, *Bacillus*, *Chitinimonas*, *Chryseobacterium*, *Flavobacterium*, *Herbaspirillum*, *Novosphingobium*, *Pseudomonas*, *Roseomonas*, *Sphingobacterium*, *Sphingomonas*, *Staphylococcus*, *Stenotrophomonas*, *Shewanella*, *Undibacterium* and *Variovorax*. No putative *Bd*‐inhibitory zOTUs were detected in the top five taxa with high relative abundance in water samples.

Neither treatment nor offspring type had a significant effect on the total number of zOTUs shared between adult males and offspring (Anova.glmmTMB: Treatment *X* ^2^ = 1.10, df = 2, *p* = 0.577; Offspring *X* ^2^ = 0.60, df = 1, *p* = 0.440; Table S7). There were no significant differences in the proportion of putative *Bd*‐inhibitory zOTUs shared between adult males and offspring across treatments (Anova.glmmTMB *X* ^2^ = 0.83, df = 2, *p* = 0.659) or offspring type (Anova.glmmTMB *X* ^2^ = 0.00, df = 1, *p* = 0.998). There were also no significant differences in the proportion of putative *Bd*‐inhibitory zOTUs acquired from biological compared to foster fathers (Anova.glmmTMB *X* ^2^ = 0.11, df = 1, *p* = 0.736) in both tadpoles and metamorphs (Anova.glmmTMB *X* ^2^ = 0.27, df = 1, *p* = 0.606; Figure 4).

**FIGURE 4:**
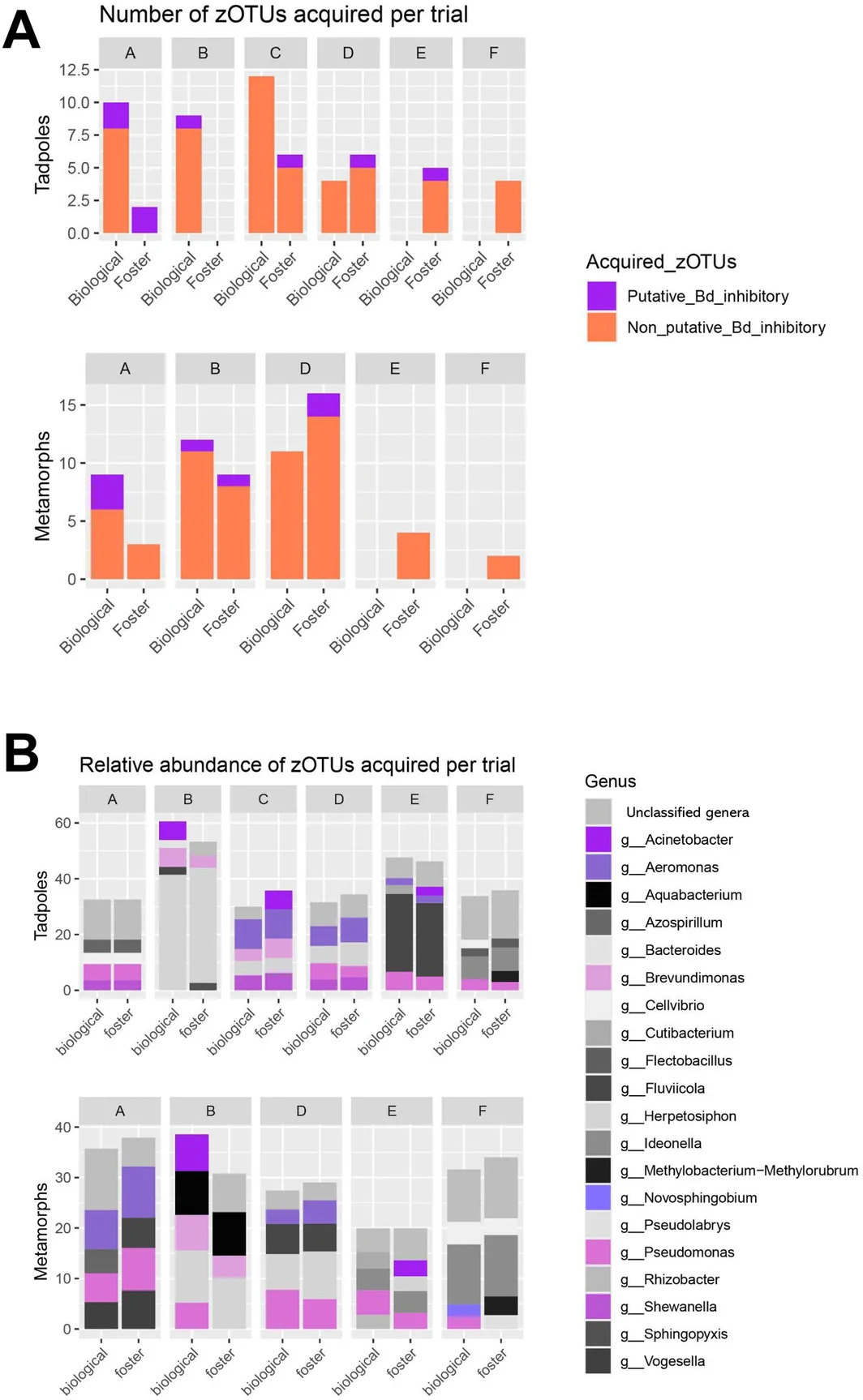
Acquired taxa per trial in the foster transport treatment. (A) Number of taxa acquired from biological and foster fathers in corresponding offspring samples (i.e., tadpoles and metamorphs). (B) Relative abundance of the acquired taxa from biological and foster fathers in corresponding offspring samples (i.e., tadpoles and metamorphs). Blocks in scales of purple and pink correspond to putative *Bd*‐inhibitory genera. Letters A–F refer to respective trials.

## 4 Discussion

Using a cross‐fostering experiment in the Neotropical poison frog *A. femoralis* , we investigated how paternal care contributes to offspring skin microbiota. Parental microbial contributions were detectable beyond metamorphosis, and some parent‐offspring shared zOTUs belonged to genera containing documented *Bd*‐inhibitory isolates. These findings support a role for parental care in microbial community assembly, while the persistence and protective functions of individual taxa remain to be tested.

Maternal microbial signatures were detectable in clutch, tadpole and metamorph samples across treatments, despite removal of females after oviposition. This pattern is consistent with microbial seeding during oviposition, although it does not establish the route of transfer or continuous persistence of individual taxa. In poison frogs, the vitelline membrane has been described as a barrier to microorganismal exchange between embryos and the jelly, leading to major colonization of microbial communities after hatching (Fischer et al. 2025). However, upon hatching, embryos immediately come into contact with the jelly matrix until the caring parent (male) collects the hatchlings. This interaction exposes the hatchlings to the jelly‐associated microbiota, initiating colonization of the tadpole skin (Fischer et al. 2025). These newly inoculated microbes have the potential to then become stable colonizers on the offspring skin, which is also supported by our study.

Similar to mothers, biological fathers seem to contribute to the microbial seeding already during fertilization. The biological father's microbial contribution was also found in offspring in the foster transport treatment, and in the no transport treatment, and these contributions are likely retained past metamorphosis. However, a considerable amount of microbiota is also acquired during tadpole transport. In the foster transport treatment, the microbiome of the foster father did not fully replace that of the biological father, nor did the initial seeding by the biological father prevent additional microbial acquisition from the foster father. Because foster fathers had access to the clutch prior to tadpole transport, microbial transfer could have occurred before transport took place. Hence, our results suggest that parentally transmitted microbiota play both seeding and reinforcing roles in microbial community establishment (see also Renelies‐Hamilton et al. 2021; Fukami 2015).

An important source of microbial acquisition in animals is the environment (e.g., Callewaert et al. 2020; Kueneman et al. 2014; Soto‐Cortés et al. 2024; Bouchali et al. 2026). In our study, substrate and water samples also contributed considerably to the offsprings' skin microbiome. Results from the SourceTracker analyses identified considerably high proportions of unknown sourcing, possibly resulting from unsampled environmental reservoirs, taxa below the detection and filtering thresholds or algorithmic limitations of SourceTracker with compositionally similar sources. Although Sourcetracker is a quite reliable method to trace bacterial origins, due to the similarities between the parental (male and female) and substrate samples, it is not easy to fully disentangle the contribution of these two sources on the offspring samples. However, by extracting the unique zOTUs that are shared only between fathers and offspring, we were able to identify the specific effects of distinct sources. Growing evidence suggests that social interactions, including parental care, can play a key role in microbial seeding. Previous studies have demonstrated that close social interactions can even reduce the contribution of the environment through, for example, engraftment processes (amphibians: Kouete et al. 2023; McGrath‐Blaser et al. 2021; Fischer et al. 2025; fish: Tanger et al. 2024). We assume that, in our study, males transferred host‐adapted microbes to the tadpoles during close contact with clutches as well as tadpole transport.

We identified important shifts in microbial composition across offspring development. Hatching, which represents the first contact of tadpoles with the external environment, was associated with major shifts in host‐associated microbiome composition. Hatching is a critical step for microbiota acquisition in egg‐laying animals, as it exposes larvae from relatively stable embryonic conditions to novel environments. Exposure to new environmental conditions has been suggested as a strong source for novel microbial communities, potentially enhancing the establishment of parental colonizers (Renelies‐Hamilton et al. 2021). The absence of significant differences in alpha diversity between tadpoles and metamorphs suggests that overall levels of microbial richness/diversity remain relatively stable across these developmental stages. However, this stability does not necessarily imply persistence of the same microbial taxa, as compositional turnover may occur without corresponding changes in alpha diversity. The observed differences between tadpole and metamorph samples suggest that a reshaping process of the microbial community takes place during metamorphosis. Changes in microbial community composition over ontogeny are widespread among animals (e.g., humans: Wang et al. 2020; Ferretti et al. 2018; non‐human mammals: Lyons et al. 2020; birds: Hernandez et al. 2021; reptiles: Bunker and Weiss 2024; amphibians: Kueneman et al. 2014; Bataille et al. 2016; fish: Tanger et al. 2024; Sylvain and Derome 2017; insects: Körner et al. 2023; Kowallik and Mikheyev 2021; other invertebrates: Turon et al. 2024). Major shifts in environmental conditions offer numerous opportunities for colonization by new bacteria. Colonization may, however, be influenced by priority effects acting either by competition (e.g., after hatching) or facilitation (e.g., after metamorphosis) with previously acquired microorganisms (reviewed by Murphy et al. 2023; e.g., amphibians: Kueneman et al. 2022; Jones et al. 2024; McGrath‐Blaser et al. 2021; Fischer et al. 2025; fish: Berggren et al. 2023; insects: Renelies‐Hamilton et al. 2021; Sinotte et al. 2023; Chen et al. 2024). Metamorphosis in amphibians represents a major morphological and physiological transition from aquatic to terrestrial environments that translate to major changes in microbiome richness and composition (Kueneman et al. 2014). In our study, we identified a considerable reorganization of host‐associated microbial communities across development. However, the initial seeding by parents was detectable beyond metamorphosis, and tadpole transport may reinforce the maturation of the offspring skin microbiome.

Social interactions have been shown to facilitate the colonization of host‐adapted taxa (Sinotte et al. 2023) and we observed a similar effect in our study: a fraction of the acquired taxa includes bacteria previously identified as key components in the immune response against *Bd*, a globally emerged pathogenic fungus (Woodhams et al. 2023; Loyau et al. 2024). We acknowledge that our analysis was conducted at the genus level and did not identify the specific species or strains that correspond to known *Bd* inhibitory taxa. These genera may therefore also include taxa that are not specifically *Bd*‐inhibitory (Burkart et al. 2017); but see also (Kruger 2020). The acquired potentially protective bacteria persisted even past metamorphosis, though the proportions shifted, indicating succession processes founded in social interactions within the context of parental care (Hakala et al. 2022; Prest et al. 2018). Further, although *A. femoralis* is considered tolerant to *Bd* infection (Courtois et al. 2015), *Bd*‐inhibitory skin bacteria may show some functional relevance. Tolerant hosts can still experience sublethal costs of infection, and skin microbiota that reduce pathogen load could mitigate these costs, reduce transmission potential to more susceptible sympatric species, or provide enhanced protection during life stages such as the tadpole‐to‐metamorph transition when *Bd* susceptibility is typically highest, independent of adult‐stage tolerance mechanisms. Our findings underline the resilience of these potentially beneficially microbial communities during extreme developmental transitions in offspring, such as tadpoles hatching in terrestrial environments and undergoing metamorphosis (Philippot et al. 2021).

Our study was conducted under controlled laboratory conditions, which reduced variation in rearing environments among treatments. However, captivity can alter amphibian microbial communities relative to those of wild populations (Kueneman et al. 2022; Korpita et al. 2023). Hence, the specific taxonomic composition, including the identity and relative abundance of putative *Bd*‐inhibitory genera, may differ from what would be observed in wild *A. femoralis* populations, and validating these transmission dynamics under field conditions represents an important next step.

In summary, our study provides experimental evidence for transmission of skin microbiota during parental care in a poison frog, with microbial contributions detectable beyond metamorphosis. In poison frogs, tadpole transport is an obligate behaviour, critical for the survival of tadpoles hatched in terrestrial environments; by actively relocating larvae to water, parents prevent desiccation and ensure offspring survival. Our findings suggest that tadpole transport is more than just a behavioural adaptation to ensure the survival of terrestrial eggs that develop into aquatic larvae. Microbiota acquired through close parent‐offspring interactions may be sustained beyond metamorphosis, with some having even the potential to enhance offspring resilience to disease. Understanding the role of parental care in enhancing social immunity could provide key insights into the mechanisms that have led to the evolution of parental care in animals.

## Author Contributions

F.N.A.‐L.: conceptualization, data curation, formal analysis, investigation, methodology, visualization, project administration, writing – original draft preparation; writing – review and editing. R.B.: formal analysis, visualization, data curation, methodology, writing – review and editing. A.L.: conceptualization, methodology, visualization, funding acquisition, validation, writing – review and editing. D.S.S.: conceptualization, resources, methodology, validation, supervision, writing – review and editing. E.R.: conceptualization, funding acquisition, methodology, supervision, resources, writing – original draft preparation, project administration, writing – review and editing.

## Funding

This study was funded by the Swiss National Science Foundation (grants no. 310030_197921 and 310030_215049 to E.R.). D.S.S. holds the AXA Chair for functional mountain ecology and D.S.S. has received financial support by the AXA Research Fund via the project GloMEc (Global Change in Mountain Ecosystems) and D.S.S., A.L. and R.B. by the Agence Nationale de la Recherche (ANR) via the project FishME (ANR‐21‐BIRE‐0002‐01).

## Ethics Statement

All testing was approved by the Swiss Federal Food Safety and Veterinary Office (National No. 33232, Cantonal No. BE144/2020). Captive conditions were approved by the Swiss Federal Food Safety and Veterinary Office (Laboratory animal husbandry licence: No. BE4/11). We followed the guidelines laid out by the ASAB for the treatment of animals in behavioural research and Teaching (ASAB Ethical Committee and ABS Animal Care Committee 2024) and the ARRIVE guidelines (du Sert et al. 2020). The founding individuals of this population were sampled in and exported from French Guiana in compliance with all legal requirements from the responsible French authorities (DIREN: Arrêté no. 82 du 10.08.2012 and Arrêté no. 4 du 14.01.2013). Individuals used in the present study are all captive bred individuals from this original stock.

## Conflicts of Interest

The authors declare no conflicts of interest.

## Supporting information


Supplementary materials File 1:
**Figure S1:** Intersection plots. (A) Normal transport with intersection sizes. (B) No transport with intersection sizes. (C) Foster transport with intersection sizes. These combinations do not take into account per‐trial combinations of adults and their corresponding offspring.
**Figure S2:** Intersection plots per trial. (A) Normal transport. (B) No transport. (C) Foster transport.
**Table S1:** Summary of sample type read numbers. Average number of reads has been rounded.
**Table S2:** Summary of pairwise comparisons using an ANOSIM test on the calculated Bray–Curtis distances between sample type composition. We applied a Bonferroni correction to account for multiple comparisons. Significant values highlighted in bold and with an *.
**Table S3:** Pairwise comparisons of alpha‐diversity indexes Chao1, Shannon and Inverse Simpson between sample types. Significant values highlighted in bold and with an *. *W* = Kruskal–Wallis estimate.
**Table S4:** Summary of tests (ANOVA and Kruskal–Wallis) of the effect of treatment on the alpha‐diversity indexes found for offspring samples. Significant values highlighted in bold and with an *; df = degrees of freedom.
**Figure S3:** Boxplot of alpha diversity indexes of offspring samples. Alpha diversity measures (A) Chao, (B) Shannon and (C) Inverse Simpson Indexes are given for offspring samples per treatment.
**Table S5:** Summary of contribution proportion of (A) all sources in offspring (Tadpoles and Metamorphs) samples and (B) developmental stages. IQR, Inter Quartile Range.
**Table S6:** (A) ANOVA summary of Generalized Linear Mixed Model (glmmTMB) to determine differences in variables affecting contribution proportions and, (B) pairwise comparisons of source contributions. Significant *p*‐values highlighted in bold.
**Figure S4:** Plot of residual and predicted values using DHARMa package. We identified significant deviations from model assumptions (dispersion = 2.38; *p* = 0.016), however, this can be explained by the large sample size of our dataset and the sensitivity of the DHARMa test. Visual inspection of the QQ plot residuals suggest that the distribution of residuals is very close to the expected optimal values.
**Table S7:** Shared zOTUs per‐trial.

**Supplementary Material File 2:** Source Contributions per offspring type.

## Data Availability

Sequences have been deposited in the ‘Inventaire des Données de la Recherche et Environnement et Sociétés’ (InDoRES) repository, https://doi.org/10.48579/PRO/95G1BH. Code and further raw data used are available here: https://doi.org/10.5281/zenodo.24056274.
