# Supplementary material for: Parental Contributions to Offspring Skin Microbiota Remain Detectable After Metamorphosis
Source: Mol Ecol. 2026 Nov 13;35(22):e70660. doi: 10.1111/mec.70660 (PMC13768829; doi:10.1111/mec.70660)
Supplement: Supplementary file 1 [file MEC-35-e70660-s002.pdf]

Supplementary materials File 1

Parental contributions to offspring skin microbiota remain detectable after metamorphosis

Francesca N. Angiolani-Larrea, Rayan Bouchali, Adeline Loyau, Dirk S. Schmeller, Eva Ringler

Detailed methods section

Laboratory frog population – housing conditions

Individuals were kept in breeding pairs in standard (60 × 40 × 40 cm) glass terraria furnished with a coconut shelter, a perch, a plant, a water bowl, and expanded clay pebbles covered with autoclaved oak leaves. The sides were covered with Xaxim (tree fern stems) and cork mats to prevent visual contact between terraria. Light, temperature (daily cycles between 23-28°C), and hydration of tanks (rain cycles twice per day to ensure humidity close to 100%) were automatically controlled to mimic natural conditions in French Guiana. All frogs were fed twice a week with vitamin-dusted fruit flies. Tadpoles received dried *Urtica* leaves ad libitum and were kept in aquaria filled with reverse osmosis water, equipped with a standard aquarium filter (Amazonas Aqua Cleaner with activated carbon and sponge filter) and air supply.

DNA extraction PCR conditions

We used the KAPA HiFi DNA Polymerase Kit for the PCR steps. In the first step PCR, conditions were: 3 minutes at 95º C, 20 seconds at 95ºC, 15 seconds at 54ºC, 15 seconds at 72ºC and 5 minutes at 72ºC in triplicates for each sample, which were pooled afterwards. Given the low yield of some samples, we pooled amplifications products from up to three PCR runs before sequencing. We used Beckam Coulter Life Sciences Agencourt AMPure XP Beads (Indianapolis, Indiana, USA) to retrieve clean DNA from the PCR product. In the second PCR step, we used the Illumina Nextera XT DNA library preparation Kit (San Diego, CA, USA) following the kit's protocol specification and 55ºC as annealing temperature. Amplicons were pooled, cleaned with beads, and sequenced on an Illumina MiSeq instrument at the GDC.

Bioinformatics

Data analyses were conducted in R 4.4.0 (R Core Team 2024), with RStudio 2024.04.2 (RStudio Team 2024). To determine the samples to be used in downstream analyses, we first we selected the adult samples with higher sequence number corresponding to the same individual. Then, we calculated the sequence counts per sample along with the percentage of sequencing coverage and validating the threshold that covers 90% of samples with the Goods coverage index (700 seq.) using the "tidyverse" package (version 2.0.0; (Wickham *et al.* 2019) and discarded samples with lower sequencing number. Additionally, we rarefied all samples to the selected threshold with the *rarefy* function from the "vegan" package (version 2.7-3 (Oksanen *et al.* 2026)). Given that a considerable proportion of the samples did not reach plateau visually, we calculated the slopes of all curves using the *rareslope* function of "vegan". To determine the best balance between number of samples available for analyses and quality of the samples, we computed sample type differences with three datasets corresponding to < 0.40, < 0.25 and < 0.1 slopes. We found that the dataset corresponding to < 0.1 slope did not have enough samples to compute any downstream analyses as key sample types were eliminated from all trials. As for < 0.40 and < 0.25 slope datasets, we found highly similar results for sample type differences (https://github.com/francescaangiol/Microbiome_Transfer_to_metamorphs). Therefore, to increase sample number, we kept <0.40 slope dataset for all downstream analyses.

We examined contamination from our samples with the package "decontam" version 1.24.0 identifying contaminants with a threshold of 0.1 using negative control samples as reference (Davis *et al.* 2018). We detected 0.12% of contamination corresponding to 27 zOTUs which were removed from the dataset.

To determine differential compositions between dorsal and ventral samples from the same frogs, we used functions from the "vegan" package. We performed a Nonmetric Multidimensional Scaling with Stable Solution from Random Starts (*metaMDS* function) paired with an ANOSIM test (*anosim* function). We used the rarefied samples and Bray-Curtis distances using 999 permutations (*rarefy* and *vegdist* functions). We did not find compositional differences between ventral and dorsal samples of the same adult frogs (ANOSIM = -0.01; p = 0.389), therefore, in rare cases (N = 5) we replaced missing dorsal samples with their ventral counterparts.

After quality control, our data set was composed of 265 samples and we had a total of 12 619 079 reads with an average of 47 619 (min: 566; max: 1 855 380) reads clustered into 6 236 zOTUs (See Table S1 for a summary of reads for all sample types). We first built a phyloseq object, using the package "phyloseq" (version 1.48.0; (McMurdie & Holmes 2013)) and the *tax_fix* function in "MicroViz" (version 0.12.3; (Barnett *et al.* 2021)) to fill empty taxonomic information with the higher known taxonomic rank. We used

zero-radius Operational Taxonomic Units (zOTUs) for all of our analyses and zOTUs belonging to non-determined eukaryotic (N = 2), mitochondria family (N = 11), chloroplast order (N = 32) and Archaea taxa (N = 18) were removed. We also discarded zOTUs with less than 700 reads.

We obtained valid microbiome information of 23 adult males, 25 adult females, 22 clutches in the first week of development, 25 clutches in the second week of development, 85 tadpoles, 55 metamorphs, 9 substrate (leaves and pebbles), and 21 water (bowl) samples.

Statistical analyses

To reduce the effect of variation in sequencing depth, we rarefied the dataset to a minimum sample depth of 700 reads and used this for all downstream analyses. A total of 2247 zOTUs were removed after random subsampling. To determine differential compositions between all sample types (beta-diversity), we normalized zOTU abundances by Hellinger method using the *decostand* function from "vegan" and visualized it with a metaMDS using Euclidean distances and 100 runs. We did an Analysis of Similarity (ANOSIM) on the calculated Bray-Curtis distances to test for overall differences between sample types with the function *anosim* ("vegan" package) and 999 permutations. Consecutively, we did pairwise comparisons with the function *anosim.pairwise* ("VeganEx" package, version 0.1.0; (ZhonghuiGai)) and 999 permutations. We applied a Bonferroni correction to account for multiple comparisons (Table S2).

To test for differences in microbial alpha-diversity between sample types across treatments, we computed Shannon, Chao1, and Inverse Simpson indexes ("phyloseq" package function *estimate_richness;* version 1.48.0 (McMurdie & Holmes 2013)) and performed an Anova (function *aov* of basic R) or a Kruskal-Wallis test (function *kruskal.test* from the package "phyloseq"), depending on the distribution of the index values. If the Kruskal-Wallis test revealed significant differences between groups, we performed pairwise comparisons (function *pairwise_wilcox_test* from the package "phyloseq"). We applied a Bonferroni correction to account for multiple comparisons.

To trace microbial acquisition from the various sources to offspring, we used the Bayesian source-sink tracking tool SourceTracker2 (Knights *et al.* 2011). We performed all SourceTracker analyses with the default parameters (rarefaction = 1 000 reads; burn-in = 100; restart = 10) and added both sink_rarefaction_depth and source_rarefaction_depth parameters set to 700 to maintain the standard rarefaction depths in all analyses. We ran the analysis three times to account for stochastic effects. We included the corresponding biological father, foster father (in the case of foster transport treatment), mother, substrate and water as sources, and tadpoles and metamorphs as developmental stages of offspring as a sink. This analysis was conducted separately for each trial. We calculated the median and interquartile ranges for sinks (Supplementay Material SM2). To test for differences in the percentage of contribution of

each source between experimental conditions, we used a generalized linear mixed effects model using the *glmmTMB* function ("glmmTMB" package, version 1.1.10 (Magnusson *et al.* 2021)) and a beta distribution with proportion of contribution from sources as response variable, the interaction between treatment, sink and source as fixed, and trial identity as random effect.

To determine the exact acquisition through tadpole transport, we calculated the zOTUs shared between fathers and tadpoles per trial from each experimental condition (normal transport, no transport, foster transport). For this, we calculated, for each trial separately, the coincidences by presence/absence in the zOTUs of all samples and extracted the ones present only in both offspring and male samples, considering offspring type and father type. We compared the number of shared zOTUs with the intersection analyses to confirm correspondence (see Figure S2). We tested the differences in the number of acquired zOTUs by applying a generalized linear mixed effects model using the *glmmTMB* function with negative binomial distribution. Number of shared zOTUs was used as response variable, treatment and offspring type were set as fixed factors and trial was used as random factor.

To closely follow the changes in skin microbial composition throughout development, we calculated the proportional contribution from one developmental stage to the next using SourceTracker. We defined three steps: 1) Embryonic development, where clutch week 1 was used as source and clutch week 2 as sink; 2) Hatching, where clutch week 2 was used as source and tadpoles as sink; and lastly 3) Metamorphosis, where tadpoles were used as source and metamorphs as sink. We calculated the medians and interquartile ranges for all sinks individually, per trial and per treatment (Supplementary Material SM2). We tested the differences in the proportion of contribution by applying a generalized linear mixed effects model using the *glmmTMB* function. We used proportion of contribution from sources as response variable, and treatment and sink as fixed effects. We used trial identity as a random effect and a beta distribution.

To identify which putative *Bd* inhibitory taxa were acquired by the offspring from adults during tadpole transport, we first inspected our full microbiome dataset to a published list of taxa known to inhibit the fungus *Bd* (Loyau *et al.* 2024). We then compared the zOTUs within tadpole and metamorph samples for each treatment to this list (Supplementary Material S2). For this analysis we used only the trials that had microbiome sequence data of all associated samples, adult males, adult female, substrate and water, available (tadpoles: normal transport: N = 3; no transport: N = 4; foster transport: N = 6; metamorphs: normal transport: N = 4; no transport: N = 4; foster transport: N = 6). Additionally, to determine if the acquisition of putative *Bd* inhibitory taxa was a non-random process, we used generalized linear mixed models (*glmmTMB* function) to compare the proportion of putative Bd inhibitory shared zOTUs by treatment for tadpoles and metamorphs separately. We used the proportion of putative *Bd* inhibitor taxa within the shared zOTUs between fathers and offspring per set, as the response variable, and treatment

(normal transport, no transport, foster transport) and offspring type were set as fixed factors and trial was used as random factor. We used an ordbeta distribution for this model. Lastly, we compared the proportion of putative *Bd* inhibitor taxa shared between foster and biological fathers in the foster transport treatment. We used the proportion of putative *Bd* inhibitor taxa within the shared zOTUs between fathers and offspring per set, as the response variable, source and offspring type were set as fixed factors and trial was used as random factor. We used a beta distribution and a zero inflation parameter for this model.

All GLM and GLMM models were diagnosed using the *simulateResiduals* function in the DHARMa "package" version 0.4.7 (Hartig 2016). Additionally, we performed an Analysis of Deviance (Type II Wald chi square test) on our models to test for the significance of the independent variables using the function *gmmTMB:::Anova.glmmTMB* ("glmmTMB" package). Anova tests were followed by paired comparisons using the *emmeans* function in the "*emmeans*" package version 1.10.7 (Lenth 2025) when applicable.

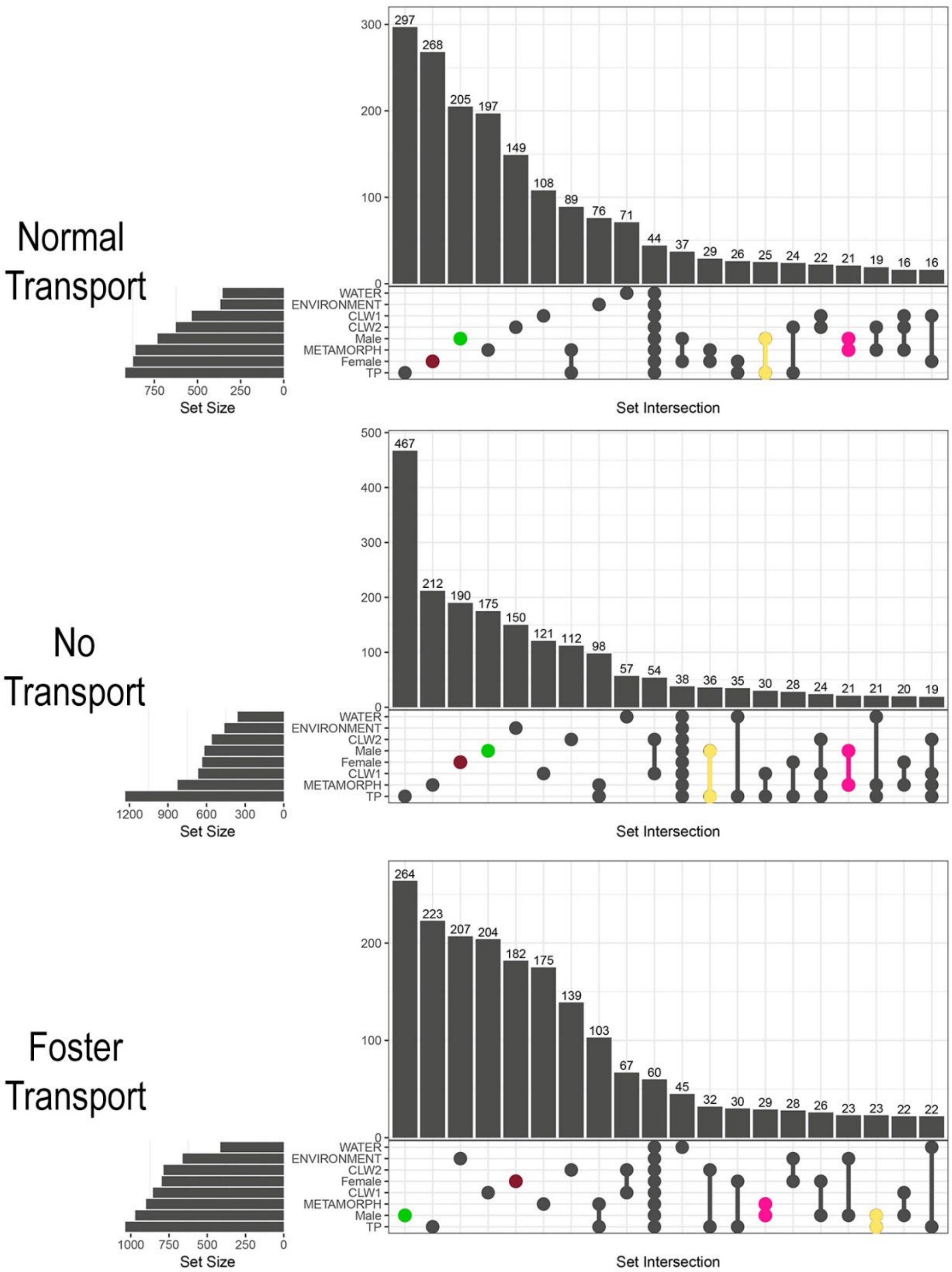

Figure S1. Intersection plots. A) Normal Transport with intersection sizes. B) No Transport with intersection sizes. C) Foster Transport with intersection sizes. These combinations do not take into account per-trial combinations of adults and their corresponding offspring.

A)

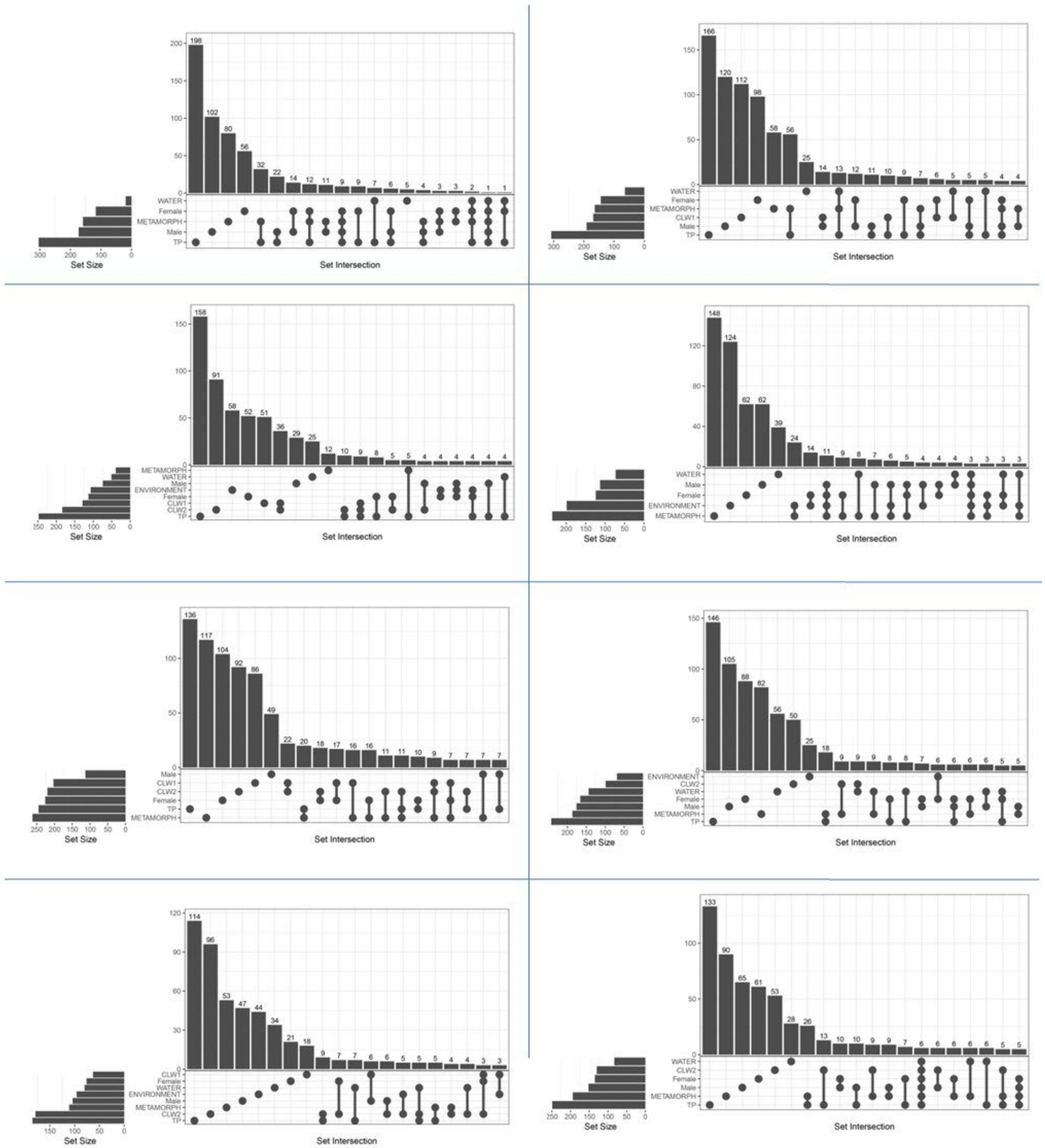

Set Size
Set Intersection
WATER
Female
METAMORPH
Male
TP
CLW1
CLW2
ENVIRONMENT

B)

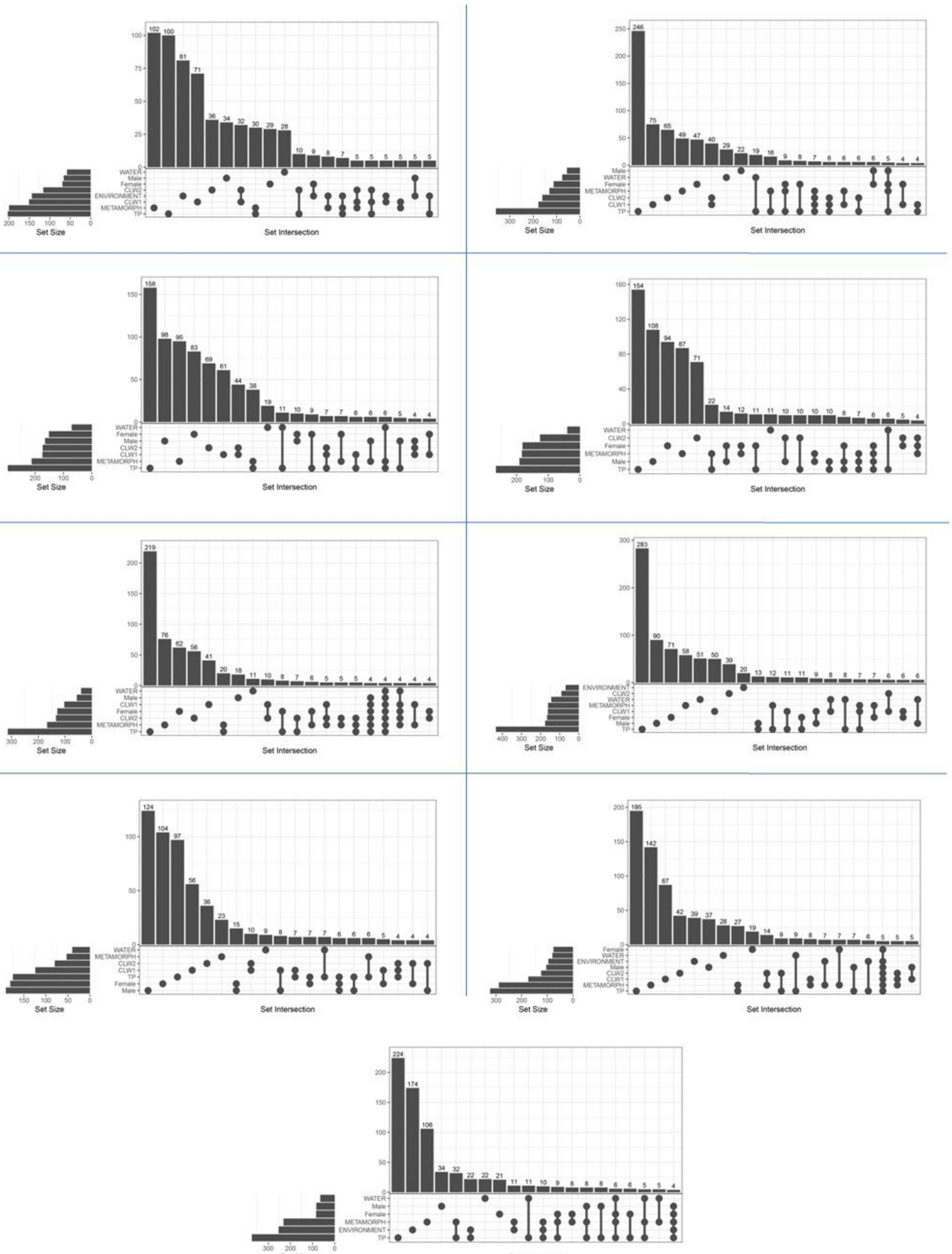

C)

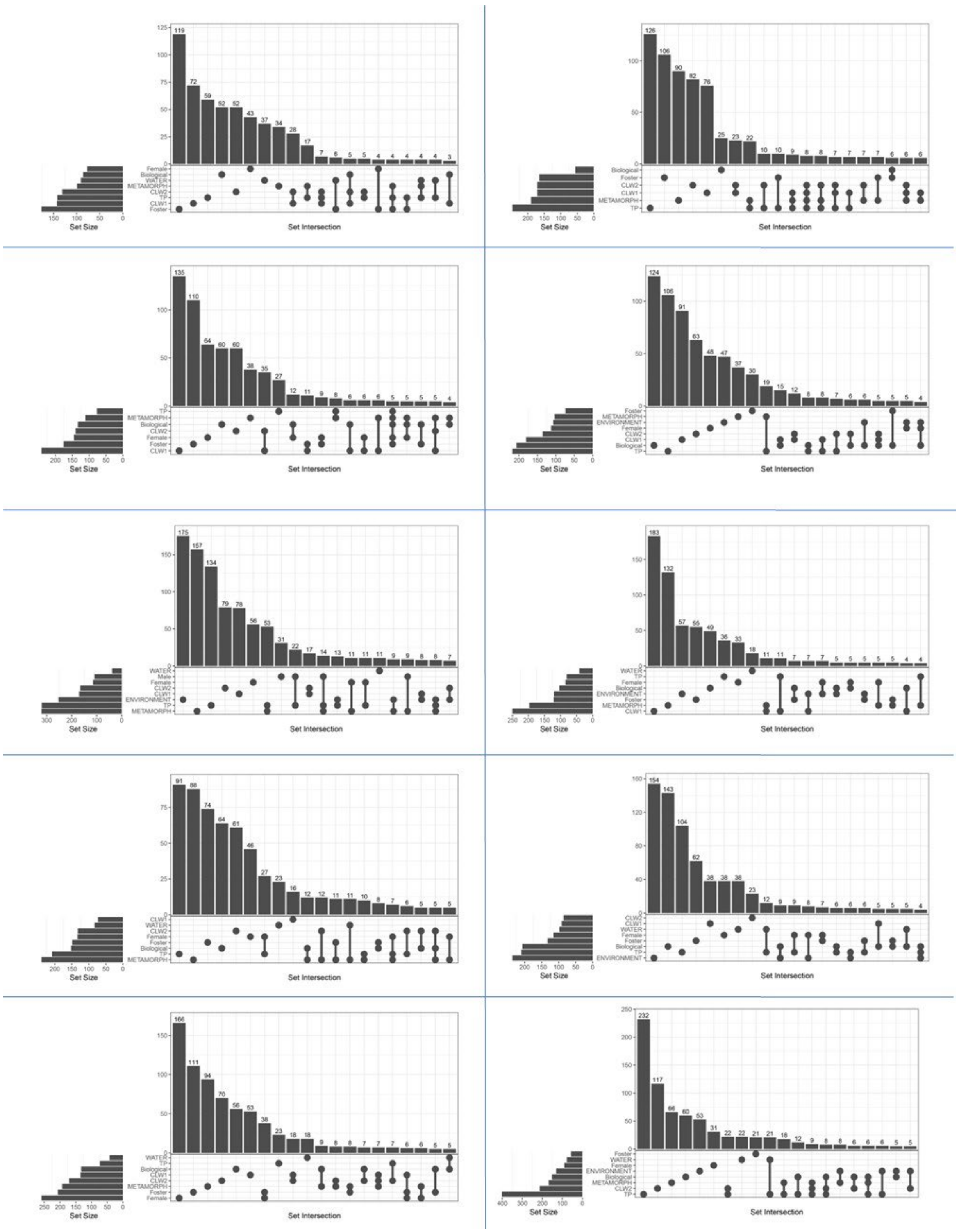

Figure S2. Intersection Plots per trial. A) Normal Transport. B) No Transport. C) Foster Transport.

Sample type composition

We found that the microbial community of all our samples was largely dominated by the phylum Proteobacteria (>50 %), followed by the phyla Bacteriodota and Verrucomicrobiota in different proportions (Fig 2B; Supplementary material S4). The paternal samples were dominated by Proteobacteria (54.19%), Actinobacteriota (12.05%), Bacteroidota (11.60%), Firmicutes (4.78%) and Verrucomicrobiota (4.39%). The maternal samples were dominated by most of the same phyla as the father, but in different proportions: Proteobacteria (51.97 %), Actinobacteriota (12.93%), Bacteroidota (12.00%), Verrucomicrobiota (4.78%) and Chloroflexi (3.75%). For the dominant taxa of the substrate samples we found: Proteobacteria (49.23%), Bacteroidota (13.27%), Actinobacteriota (12.27%), Chloroflexi (4.87%) and Verrucomicrobiota (4.02%). Water samples were largely dominated by the phyla Proteobacteria (83.16 %), and in lower proportions by Bacteroidota (9.52 %), Verrucomicrobiota (1.52 %), Actinobacteriota (1.30 %) and Planctomycetota (0.97%). Offspring samples were dominated by Proteobacteria (w1 54.91%; w2 43.93%; tadpoles 63.46%; metamorphs 62.85%), Bacteroidota (w1 20.70%; w2 18.69%; tadpoles 13.36%; metamorphs 20.34%); Verrucomicrobiota (w1 10.27%; w2 21.61%; tadpoles 4.76%; metamorphs 4.40%), Actinobacteriota (w1 3.86%; metamorphs 3.65%), Bdellovibrionota (w1 2.81%; w2 2.29%), Planctomycetota (w2 4.25%; tadpoles 4.15%; metamorphs 1.84%), and Chloroflexi (tadpoles 5.04%). Across all samples, we found several taxa that have been previously identified as inhibitory for *Bd.* The genus *Aeromonas* was among the dominant taxa in samples of males (3.89 %), females (3.39 %),tadpoles (9.75 %), metamorphs (6.59 %) and substrate (5.30%); the genus *Pseudomonas* was very abundant among males (5.32 %), females (6.37 %), clutches (W1=5.77 %), tadpoles (4.08 %), metamorphs (6.81 %) and substrate (7.38 %); the genus *Shewanella* was among the dominant taxa in tadpole samples (6.80 %), and the genera *Pedobacter* were found to be very abundant in the early (4.26 %) and late clutch samples (2.61 %), *Sphingomonas* in the early clutch (2.83 %), and *Flavobacterium* (2.28 %) in the late clutch samples. We did not find any taxa known to be inhibitory of *Bd* to be dominant in the water samples. (Details in https://github.com/francescaangiol/Microbiome_Transfer_to_metamorphs).

Dominant taxa are considered to be within the top 5 taxa with higher relative abundance.

Table S1. Summary of sample type read numbers. Average number of reads has been rounded.

Sample type	Number of samples	Number of reads	Average number of reads	Minimum number of reads	Maximum number of reads	zOTUs
Males	23	853054	37089	2981	300510	6236
Females	25	1452805	58112	973	555021	6236
Clutch Week 1	22	2769535	125888	5377	587246	6236
Clutch Week 2	25	1684992	67400	5982	274984	6236
Tadpoles	85	962744	11326	1120	159488	6236
Metamorphs	55	811062	14747	566	196019	6236
Substrate	9	64616	7180	5160	9882	6236
Water	21	4020271	191441	2026	1855380	6236

Table S2. Summary of pairwise comparisons using an ANOSIM test on the calculated Bray-Curtis distances between sample type composition. We applied a Bonferroni correction to account for multiple comparisons. Significant values highlighted in bold and with an *.

Pairs	anosimR	*p*-value	p-adj
Male.vs.Female	-0.02	0.881	1
Male.vs.Substrate	-0.11	0.848	1
Male.vs.Clutch_W1	0.50	0.001	**0.028***
Male.vs.Water	0.67	0.001	**0.028***
Male.vs.Clutch_W2	0.75	0.001	**0.028***
Male.vs.Tadpole	0.77	0.001	**0.028***
Male.vs.Metamorph	0.42	0.001	**0.028***
Female.vs.Substrate	-0.12	0.887	1
Female.vs.Clutch_W1	0.43	0.001	**0.028***
Female.vs.Water	0.64	0.001	**0.028***
Female.vs.Clutch_W2	0.72	0.001	**0.028***
Female.vs.Tadpole	0.73	0.001	**0.028***
Female.vs.Metamorph	0.37	0.001	**0.028***
Substrate.vs.Clutch_W1	0.62	0.001	**0.028***
Substrate.vs.Water	0.74	0.001	**0.028***
Substrate.vs.Clutch_W2	0.91	0.001	**0.028***
Substrate.vs.Tadpole	0.81	0.001	**0.028***

Substrate.vs.Metamorph	0.37	0.002	0.056
Clutch_W1.vs.Water	0.70	0.001	**0.028***
Clutch_W1.vs.Clutch_W2	0.16	0.002	0.056
Clutch_W1.vs.Tadpole	0.87	0.001	**0.028***
Clutch_W1.vs.Metamorph	0.47	0.001	**0.028***
Water.vs.Clutch_W2	0.79	0.001	**0.028***
Water.vs.Tadpole	0.80	0.001	**0.028***
Water.vs.Metamorph	0.56	0.001	**0.028***
Clutch_W2.vs.Tadpole	0.90	0.001	**0.028***
Clutch_W2.vs.Metamorph	0.65	0.001	**0.028***
Tadpole.vs.Metamorph	0.50	0.001	**0.028***

Alpha diversity results

When looking at overall alpha-diversity, we found significant differences between sample types (Kruskal-Wallis, Chao1 X2 = 66.91, df = 7, $p < 0.01$; Shannon, X2 = 77.51, df = 7, $p < 0.01$; Inverse Simpson X2 = 91.33, df = 7, $p < 0.01$). Therefore, we explored pairwise comparisons between sample types (Table S3).

Table S3. Pairwise comparisons of alpha-diversity indexes Chao1, Shannon and Inverse Simpson between sample types. Significant values highlighted in bold and with an *. W = Kruskal-Wallis estimate.

**Chao1**														
	**Males**		**Females**		**Substrate**		**Clutches_w1**		**Clutches_w2**		**Tadpoles**		**Metamorphs**	
	W	*p-adj*	W	*p-adj*	W	*p-adj*	W	*p-adj*	W	*p-adj*	W	*p-adj*	W	*p-adj*
**Males**														
**Females**	345	1												
**Substrate**	129	1	117	1										
**Clutches_W1**	428	**<0.001***	430	**0.019***	144	1								
**Clutches_W2**	503	**<0.001***	514	**0.001***	168	0.838	289	1						
**Tadpoles**	582	0.085	778	1	326	1	1458	**0.002***	1703	**<0.001***				
**Metamorphs**	553	1	706	1	273	1	1001	**<0.001***	1181	**<0.001***	1734	0.282		
**Water**	302	1	376	0.328	138	1	427	**<0.001***	504	**<0.001***	1499	**<0.001***	793	0.350
**Shannon**														
	**Males**		**Females**		**Substrate**		**Clutches_w1**		**Clutches_w2**		**Tadpoles**		**Metamorphs**	
	W	*p-adj*	W	*p-adj*	W	*p-adj*	W	*p-adj*	W	*p-adj*	W	*p-adj*	W	*p-adj*
**Males**														
**Females**	353	1												
**Substrate**	139	1	125	1										
**Clutches_W1**	247	1	193	1	66	1								
**Clutches_W2**	216	1	155	0.052	53	0.539	339	1						
**Tadpoles**	1359	0.119	1751	**<0.001***	632	**0.039***	1262	0.332	1185	1				
**Metamorphs**	877	0.210	1125	**<0.001***	408	0.056	845	0.194	818	1	2157	1		
**Water**	441	**<0.001***	515	**<0.001***	185	**<0.001***	433	**<0.001***	469	**<0.001***	1548	**<0.001***	954	**<0.001***
**Inverse Simpson**														
	**Males**		**Females**		**Substrate**		**Clutches_w1**		**Clutches_w2**		**Tadpoles**		**Metamorphs**	
	W	*p-adj*	W	*p-adj*	W	*p-adj*	W	*p-adj*	W	*p-adj*	W	*p-adj*	W	*p-adj*
**Males**														
**Females**	332	1												
**Substrate**	113	1	105	1										
**Clutches_W1**	172	1	117	**0.022***	46	0.568								
**Clutches_W2**	130	**0.024***	66	**<0.001***	26	**0.009***	342	1						
**Tadpoles**	1511	**0.002***	1888	**<0.001***	669	**<0.001***	1197	**0.044***	1080	1				
**Metamorphs**	968	**0.007***	1190	**<0.001***	425	**0.018***	768	1	716	1	2288	1		
**Water**	446	**<0.001***	522	**<0.001***	189	**<0.001***	428	**<0.001***	443	**<0.001***	1509	**<0.001***	955	**<0.001***

Table S4. Summary of tests (ANOVA and Kruskal-Wallis) of the effect of treatment on the alpha-diversity indexes found for offspring samples. Significant values highlighted in bold and with an *; df = degrees of freedom.

**Offspring sinks**	**Test type**	**Estimate**	**df**	**p-value**
Clutches w1				
Chao1	Anova	$F = 0.09$	2	0.914
Shannon	Anova	$F = 0.02$	2	0.977
Inverse Simpson	Kruskal-Wallis	$X^2 = 0.07$	2	0.964
Clutches w2				
Chao1	Anova	$F = 2.44$	2	0.111
Shannon	Anova	$F = 2.34$	2	0.12
Inverse Simpson	Anova	$F = 3.14$	2	0.063
Tadpoles				
Chao1	Kruskal-Wallis	$X^2 = 0.85$	2	0.654
Shannon	Kruskal-Wallis	$X^2 = 4.44$	2	0.108
Inverse Simpson	Kruskal-Wallis	$X^2 = 4.62$	2	0.099
Metamorphs				
Chao1	Anova	$F = 1.83$	2	0.171
Shannon	Kruskal-Wallis	$X^2 = 0.85$	2	0.655
Inverse Simpson	Kruskal-Wallis	$X^2 = 0.28$	2	0.870

Chao1:

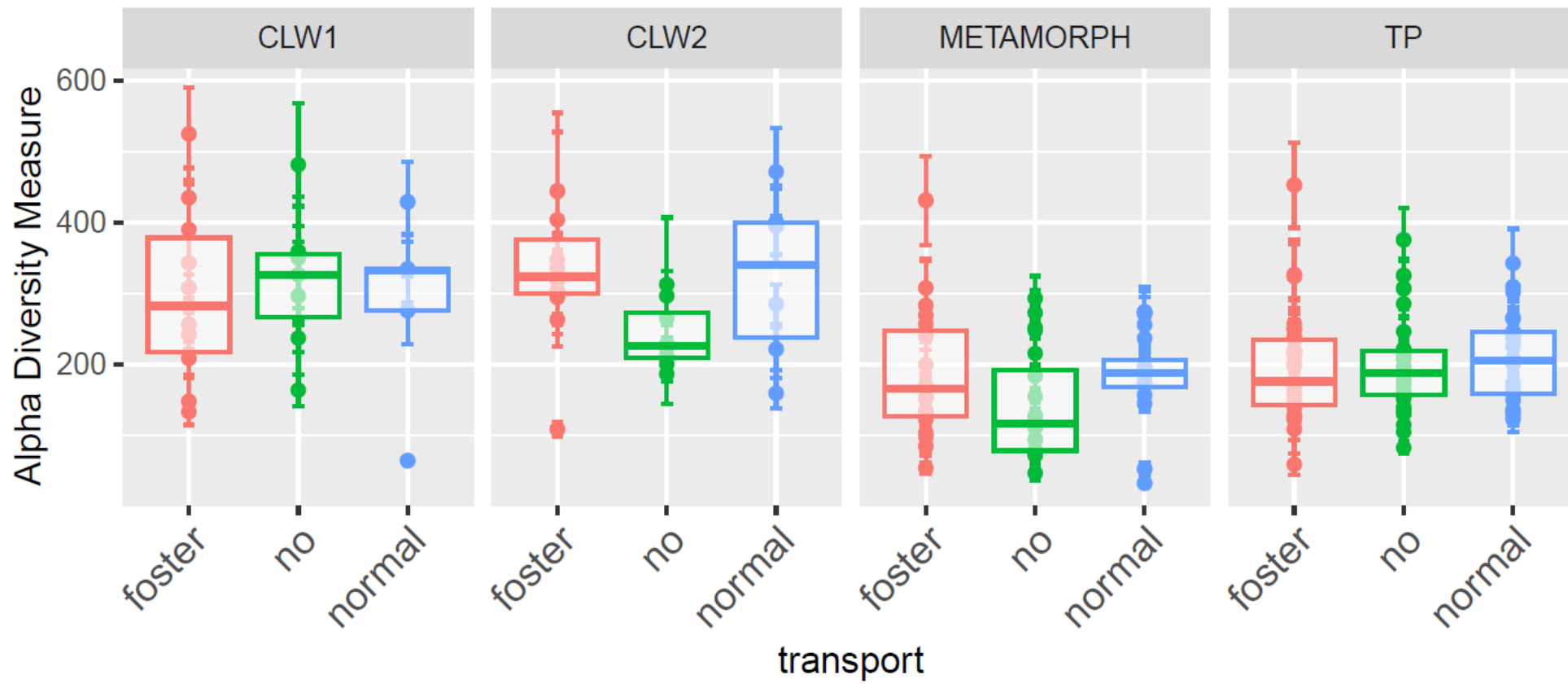

Shannon:

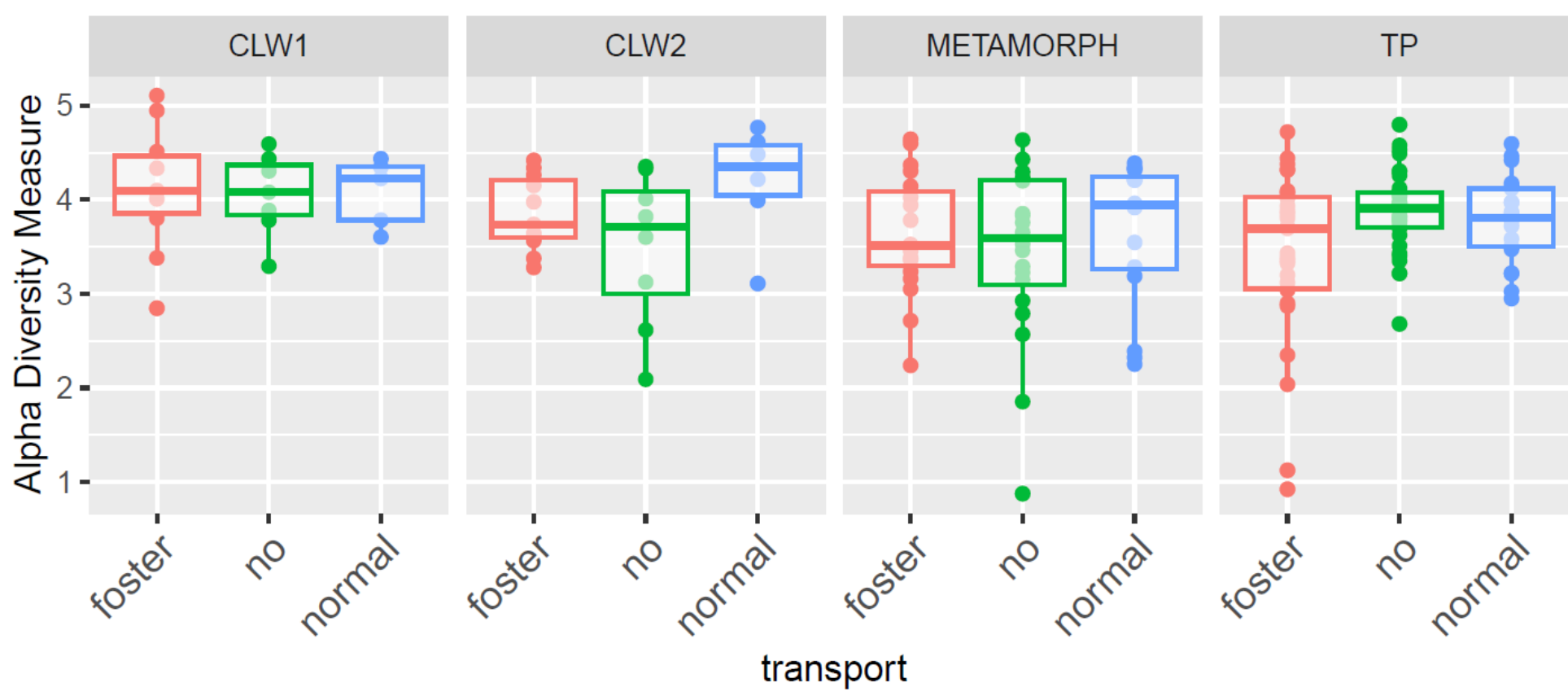

Inverse Simpson:

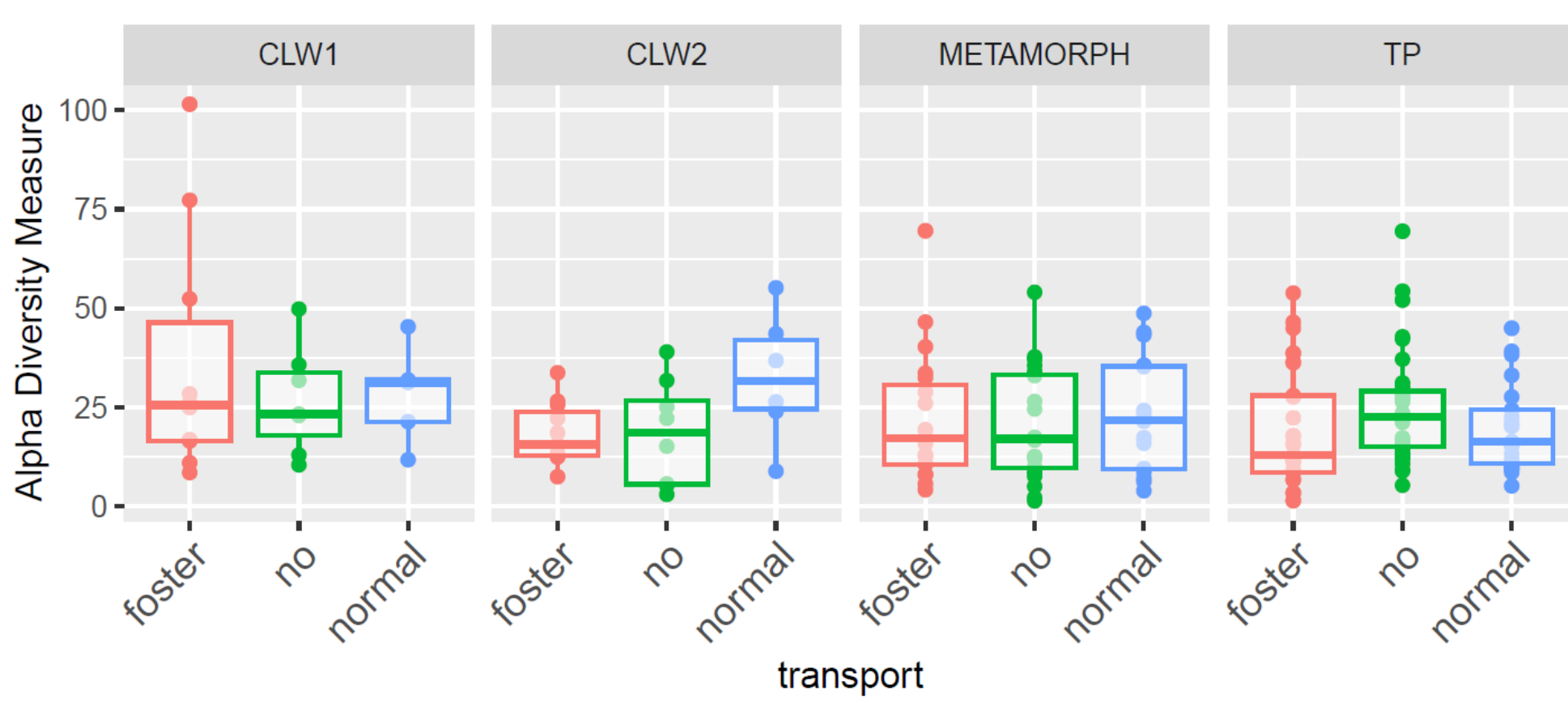

Figure S3. Boxplot of alpha diversity indexes of offspring samples. Alpha diversity measures A) Chao, B) Shannon, and C) Inverse Simpson Indexes are given for offspring samples per treatment.

Table S5. Summary of contribution proportion of A) all sources in offspring (Tadpoles and Metamorphs) samples, and B) developmental stages. IQR = Inter Quartile Range

A) Sourcing				
**Treatment**	**Source**	**Offspring**	**Contribution median**	**IQR**
Normal Transport	Biological Father	Tadpoles	0.008	0.013178571
		Metamorphs	0.013285714	0.0055
	Female	Tadpoles	0.020857143	0.024392857
		Metamorphs	0.012857143	0.0145
	Substrate	Tadpoles	0.0225	0.004035714
		Metamorphs	0.021571429	0.0175
	Water	Tadpoles	0.012571429	0.008107143
		Metamorphs	0.020428571	0.025428571
	Unknown	Tadpoles	0.922214286	0.041785714
		Metamorphs	0.911285714	0.058428571
No Transport	Biological Father	Tadpoles	0.010357143	0.013821429
		Metamorphs	0.01	0.026785714
	Female	Tadpoles	0.015571429	0.018107143
		Metamorphs	0.043857143	0.073571429
	Substrate	Tadpoles	0.018357143	0.017821429
		Metamorphs	0.012571429	0.030714286
	Water	Tadpoles	0.032285714	0.092607143
		Metamorphs	0.043714286	0.052214286
	Unknown	Tadpoles	0.890928571	0.171321429
		Metamorphs	0.855714286	0.155071429
Foster Transport	Biological Father	Tadpoles	0.014857143	0.008142857
		Metamorphs	0.025571429	0.035714286
	Foster Father	Tadpoles	0.008857143	0.005642857
		Metamorphs	0.015142857	0.031571429
	Female	Tadpoles	0.015	0.071642857
		Metamorphs	0.013857143	0.005714286
	Substrate	Tadpoles	0.016428571	0.007178571
		Metamorphs	0.040285714	0.01075
	Water	Tadpoles	0.020285714	0.012357143
		Metamorphs	0.015	0.024571429
	Unknown	Tadpoles	0.916428571	0.097928571
		Metamorphs	0.874714286	0.072428571

Table S6. A) ANOVA summary of Generalized Linear Mixed Model (glmmTMB) to determine differences in variables affecting contribution proportions and, B) pairwise comparisons of source contributions. Significant *p*-values highlighted in bold.

A)

Parameter	X^2	df	*p*-value
Treatment	1.25	2	0.535
Source	31.75	4	**<0.001***
Offspring	1.92	1	0.166

B)

Contrasts	Estimate	SE	*z*	*p*-value
Biological_Father - Female	-0.87	0.24	-3.60	**0.003***
Biological_Father - Foster_Father	-0.40	0.31	-1.30	0.689
Biological_Father - Substrate	-0.46	0.21	-2.13	0.208
Biological_Father - Water	-1.04	0.21	-5.02	**<0.001***
Female - Foster_Father	0.465	0.34	1.36	0.655
Female - Substrate	0.410	0.29	1.43	0.611
Female - Water	-0.176	0.28	-0.63	0.971
Foster_Father - Substrate	-0.054	0.35	-0.15	0.999
Foster_Father - Water	-0.641	0.34	-1.87	0.332
Substrate - Water	-0.586	0.25	-2.31	0.141

Diagnostic plot for glmmTMB model testing differences in proportion of contribution of the known sources on the offspring

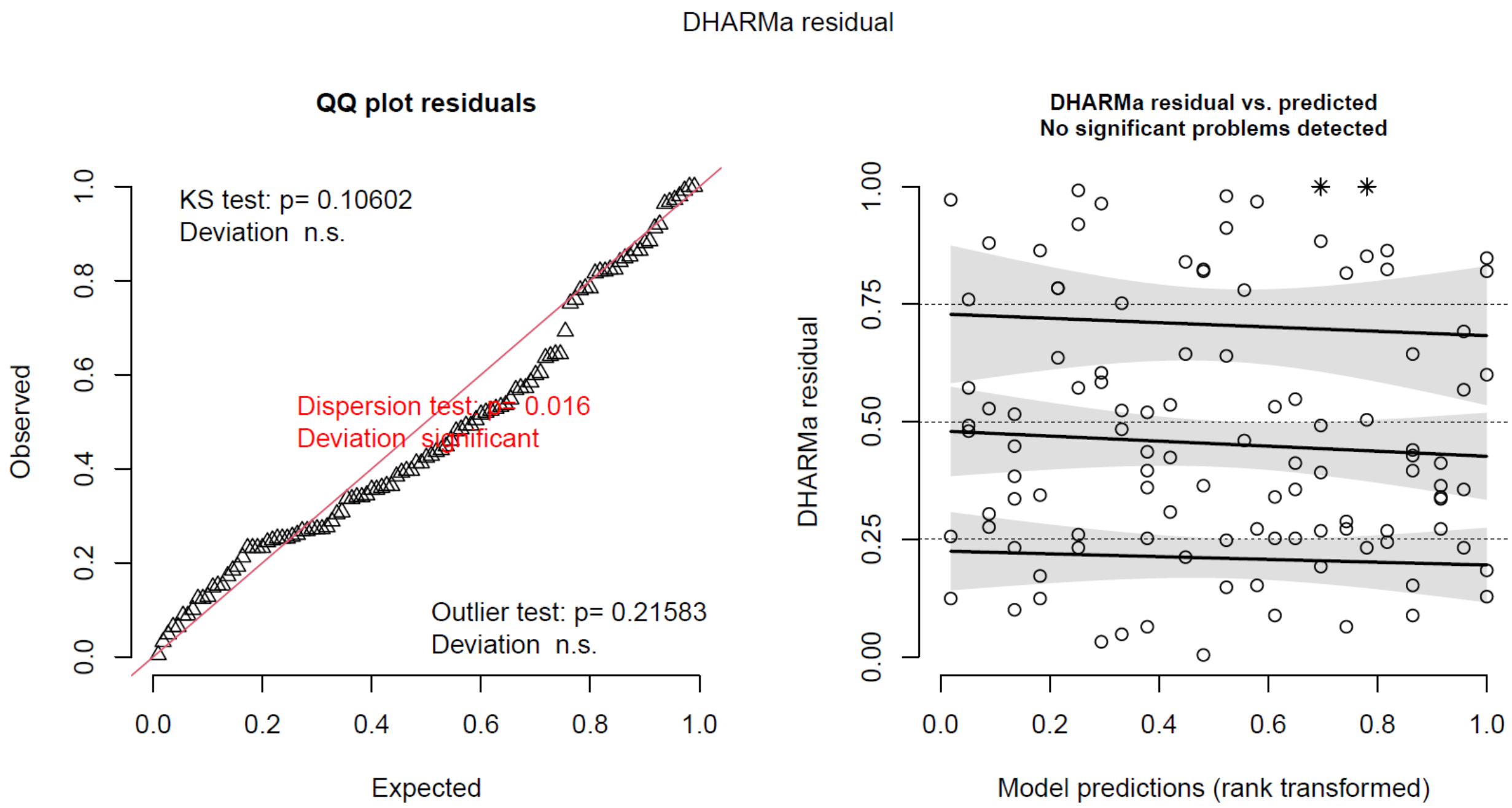

Figure S4. Plot of residual and predicted values using DHARMa package. We identified significant deviations from model assumptions (dispersion =2.38; p = 0.016), however, this can be explained by the large sample size of our dataset and the sensitivity of the DHARMa test. Visual inspection of the QQ plot residuals suggest that the distribution of residuals is very close to the expected optimal values.

Table S7. Shared zOTUs per-trial

Trial	Treatment	Source	Offspring type	Numer of zOTUs shared	Number of putative Bd inhibirtory zOTUs shared	Percentage of putative Bd inhibirtory zOTUs shared	Proportion of putative Bd inhibirtory zOTUs shared
S3	Foster_T	Foster	TP	1	0	0	0
S3	Foster_T	Foster	M	2	1	50	0.5
S3	Foster_T	Biological	TP	12	0	0	0
S3	Foster_T	Biological	M	3	2	66.66667	0.666667
S23	Foster_T	Foster	TP	3	1	33.33333	0.333333
S23	Foster_T	Foster	M	8	1	12.5	0.125
S23	Foster_T	Biological	TP	4	0	0	0
S23	Foster_T	Biological	M	8	3	37.5	0.375
S24	Foster_T	Foster	TP	6	1	16.66667	0.166667
S24	Foster_T	Foster	M	0	0	0	0
S24	Foster_T	Biological	TP	6	1	16.66667	0.166667
S24	Foster_T	Biological	M	5	1	20	0.2
S26	Foster_T	Foster	TP	3	0	0	0
S26	Foster_T	Foster	M	11	3	27.27273	0.272727
S26	Foster_T	Biological	TP	3	0	0	0
S26	Foster_T	Biological	M	12	4	33.33333	0.333333
S28	Foster_T	Foster	TP	2	0	0	0
S28	Foster_T	Foster	M	5	0	0	0
S28	Foster_T	Biological	TP	1	0	0	0
S28	Foster_T	Biological	M	2	0	0	0
S52	Foster_T	Foster	TP	4	0	0	0
S52	Foster_T	Foster	M	3	0	0	0
S52	Foster_T	Biological	TP	0	0	0	0
S52	Foster_T	Biological	M	1	0	0	0

References

Barnett D, Arts I, Penders J (2021) microViz: an R package for microbiome data visualization and statistics. *Journal of Open Source Software*, **6**, 3201, doi: 10.21105/joss.03201.

Davis NM, Proctor DM, Holmes SP, Relman DA, Callahan BJ (2018) Simple statistical identification and removal of contaminant sequences in marker-gene and metagenomics data. *Microbiome*, **6**, 226, doi: 10.1186/s40168-018-0605-2.

Hartig F (2016) *DHARMa: Residual Diagnostics for Hierarchical (Multi-Level / Mixed) Regression Models. R package version 0.4.7.* The R Foundation.

Knights D, Kuczynski J, Charlson ES *et al.* (2011) Bayesian community-wide culture-independent microbial source tracking. *Nature Methods*, **8**, 761–763, doi: 10.1038/nmeth.1650.

Lenth RV (2025) *emmeans: estimated marginal means, aka least-squares means. R package*.

Loyau A, Bouchali R, Sentenac H, Schmeller DS (2024) The commensal skin microbiome of amphibian mountain populations and its association with the pathogen *Batrachochytrium dendrobatidis*. *Environmental Microbiology*, **26**, e16699, doi: 10.1111/1462-2920.16699.

Magnusson A, Skaug H, Nielsen A *et al.* (2021) *glmmTMB: generalized linear mixed models using Template Model Builder*.

McMurdie PJ, Holmes S (2013) phyloseq: an R package for reproducible interactive analysis and graphics of microbiome census data. *PloS one*, **8**, e61217, doi: 10.1371/journal.pone.0061217.

Oksanen J, Simpson G, Blanchet F, Kindt R, Legendre P, Minchin P, O'Hara R, Solymos P, Stevens M, Szoecs E, Wagner H, Bedward M, Bolker B, Borcard D, Carvalho G, De Caceres M, Durand S, Evangelista H, Hannigan G, Hill M, Lahti L, Martino C, Ouellette M, Ribeiro Cunha E, Smith T, Stier A, Ter Braak C, Weedon J (2026). *vegan: Community Ecology Package*. R package version 2.8-0, https://vegandevs.github.io/vegan/.

R Core Team (2024) *R: a language and environment for statistical computing.* R Foundation for Statistical Computing, Vienna, Austria.

RStudio Team (2024) *RStudio: Integrated Development for R.* RStudio, Inc., Boston, MA, USA.

Wickham H, Averick M, Bryan J *et al.* (2019) Welcome to the Tidyverse. *Journal of Open Source Software*, **4**, 1686, doi: 10.21105/joss.01686.

ZhonghuiGai *VeganEx*. https://github.com/ZhonghuiGai/veganEx.
